# Supplementary material for: Efficacy and clinicogenomic correlates of response to immune checkpoint inhibitors alone or with chemotherapy in non-small cell lung cancer
Source: Nat Commun. 2023 Feb 8;14:695. doi: 10.1038/s41467-023-36328-z (PMC9908867; doi:10.1038/s41467-023-36328-z)
Supplement: Supplementary file 1 — Supplementary Information [file 41467_2023_36328_MOESM1_ESM.pdf]

Supplementary Information

Efficacy and clinicogenomic correlates of response to immune checkpoint inhibitors alone or with chemotherapy in non-small cell lung cancer

Hong, et al

Contents

Supplementary Figures. 1-22
Supplementary Table 1

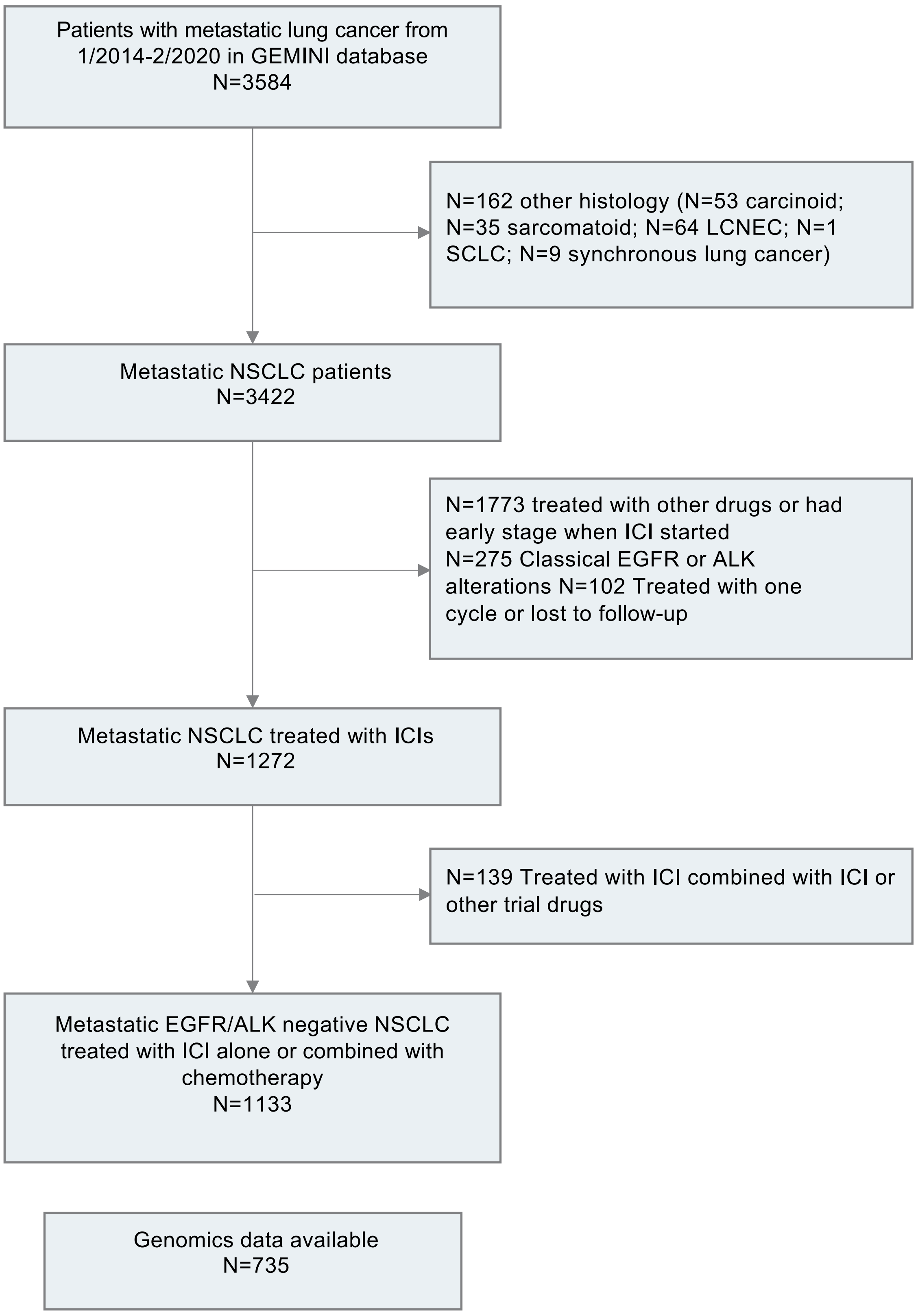
Patients with metastatic lung cancer from 1/2014-2/2020 in GEMINI database N=3584
N=162 other histology (N=53 carcinoid; N=35 sarcomatoid; N=64 LCNEC; N=1 SCLC; N=9 synchronous lung cancer)
Metastatic NSCLC patients N=3422
N=1773 treated with other drugs or had early stage when ICI started
N=275 Classical EGFR or ALK alterations N=102 Treated with one cycle or lost to follow-up
Metastatic NSCLC treated with ICIs N=1272
N=139 Treated with ICI combined with ICI or other trial drugs
Metastatic EGFR/ALK negative NSCLC treated with ICI alone or combined with chemotherapy N=1133
Genomics data available N=735

Supplementary Figure 1. **Cohort diagram**. LCNEC, large cell neuroendocrine carcinoma; SCLC, small cell lung cancer; NSCLC, non-small cell lung cancer; ICI, immune checkpoint inhibitor.

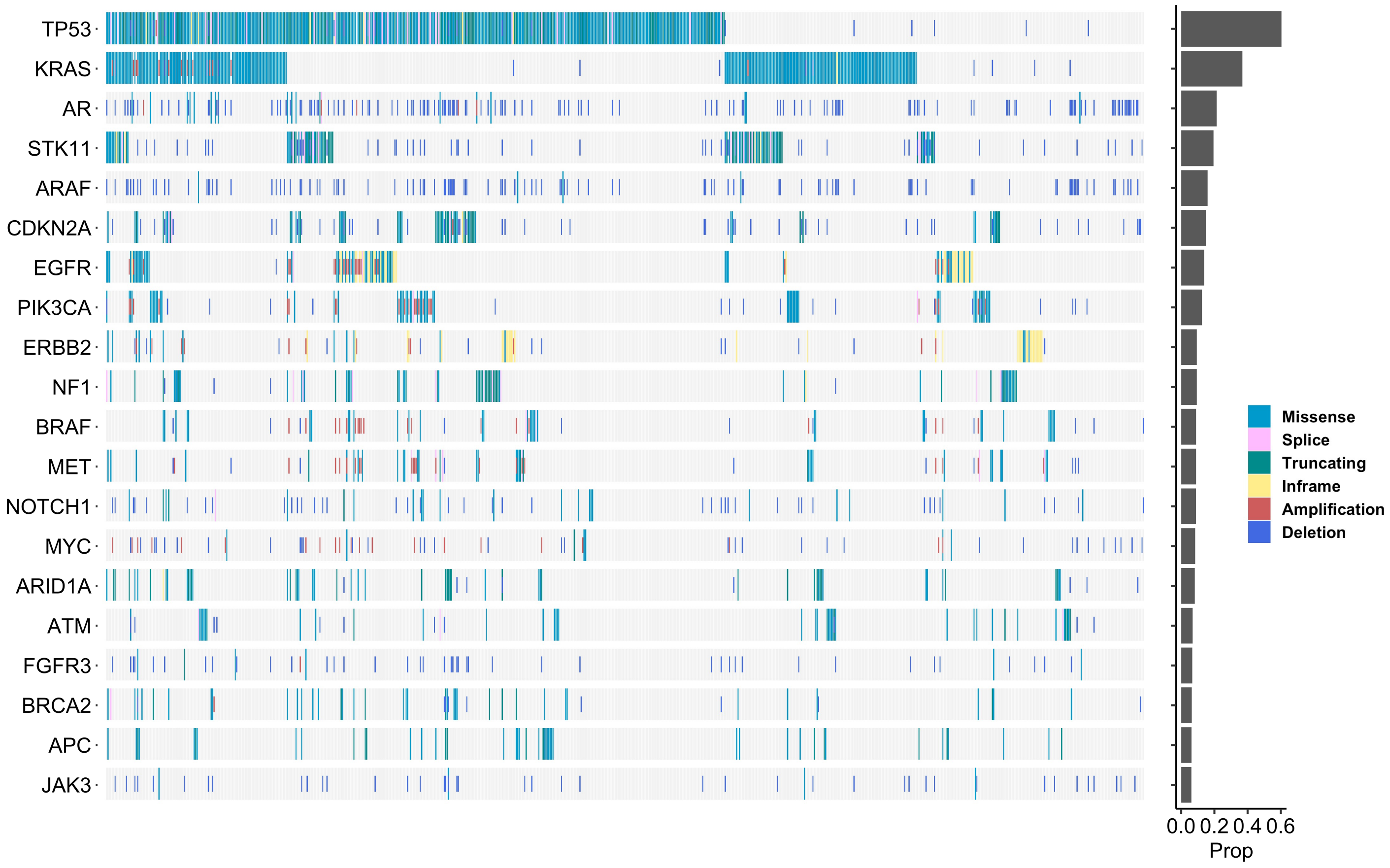
TP53
KRAS
AR
STK11
ARAF
CDKN2A
EGFR
PIK3CA
ERBB2
NF1
BRAF
MET
NOTCH1
MYC
ARID1A
ATM
FGFR3
BRCA2
APC
JAK3
0.0 0.2 0.4 0.6
Prop
Missense
Splice
Truncating
Inframe
Amplification
Deletion

Supplementary Figure 2. **Co-mutation plot depicting the mutation type and frequency of genomic alterations in the MDACC-primary cohort (n=735)**. Source data are provided as a Source Data file.

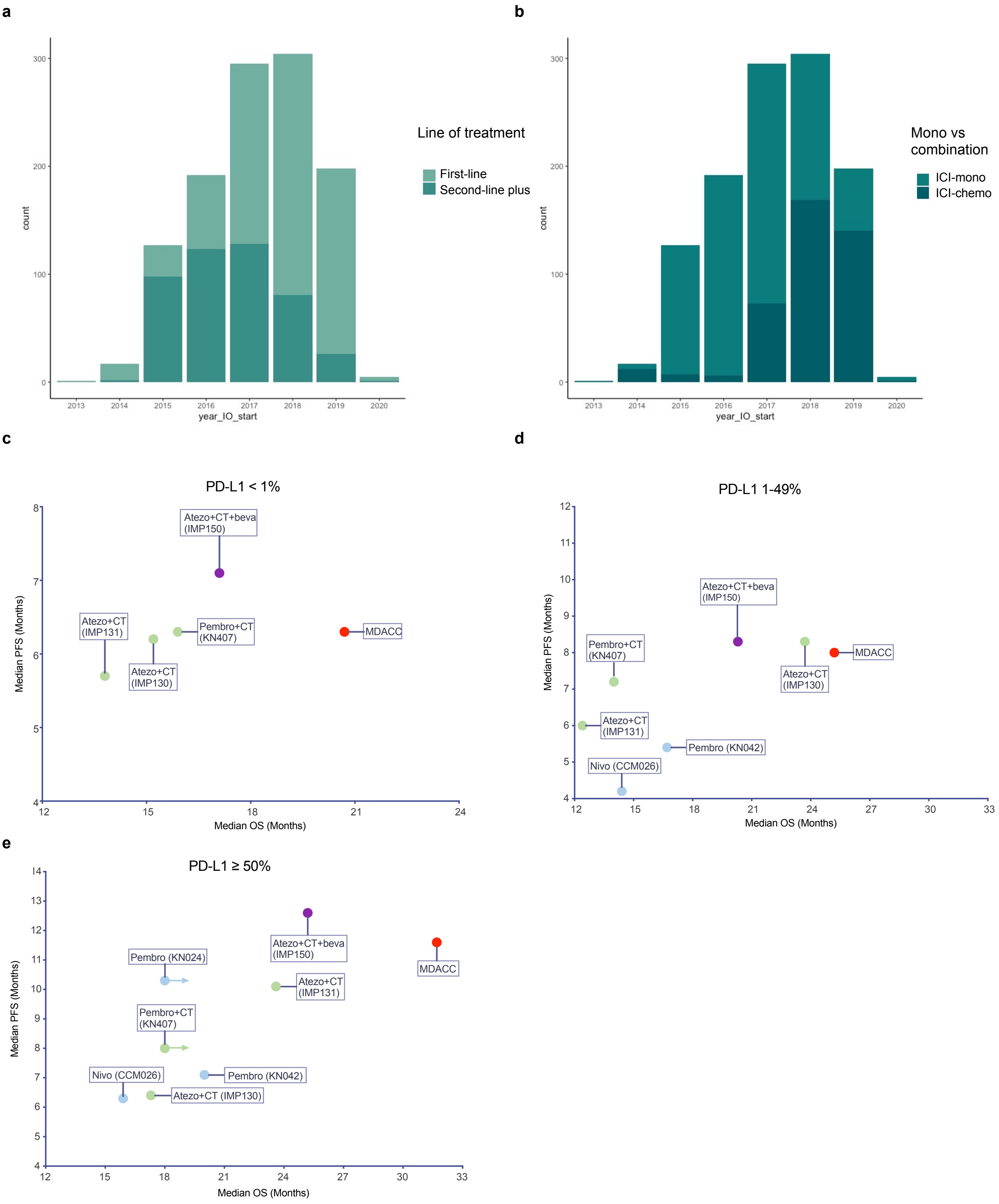
a
b
Line of treatment
First-line
Second-line plus
count
year_IO_start
Mono vs
combination
ICI-mono
ICI-chemo
count
year_IO_start
c
d
PD-L1 < 1%
Atezo+CT+beva (IMP150)
Atezo+CT (IMP131)
Atezo+CT (IMP130)
Pembro+CT (KN407)
MDACC
Median PFS (Months)
Median OS (Months)
PD-L1 1-49%
Atezo+CT+beva (IMP150)
Pembro+CT (KN407)
Atezo+CT (IMP130)
MDACC
Atezo+CT (IMP131)
Pembro (KN042)
Nivo (CCM026)
Median PFS (Months)
Median OS (Months)
e
PD-L1 ≥ 50%
Pembro (KN024)
Atezo+CT+beva (IMP150)
MDACC
Atezo+CT (IMP131)
Pembro+CT (KN407)
Nivo (CCM026)
Pembro (KN042)
Atezo+CT (IMP130)
Median PFS (Months)
Median OS (Months)

Supplementary Figure 3. **Treatment strategies over time and outcomes of immune checkpoint inhibitor (ICI) first-line setting compared to published clinical trials**. Histogram of patients treated with ICI therapy over start time (year_IO_start), colored by **a**, line of therapy and **b**, by treatment with ICI monotherapy (ICI-mono) or ICI combined with chemotherapy (ICI-chemo). Comparison of published vs MDACC-primary cohort-specific median overall survival (OS) and progression-free survival (PFS) in patients with **c,** Low PD-L1 (< 1%) treated with ICI-chemo; **d**, Intermediate PD-L1 (1-49%) treated with ICI-mono or ICI-chemo; **e**, High PD-L1 (≥ 50%) treated with ICI-mono or ICI-chemo. Source data are provided as a Source Data file.

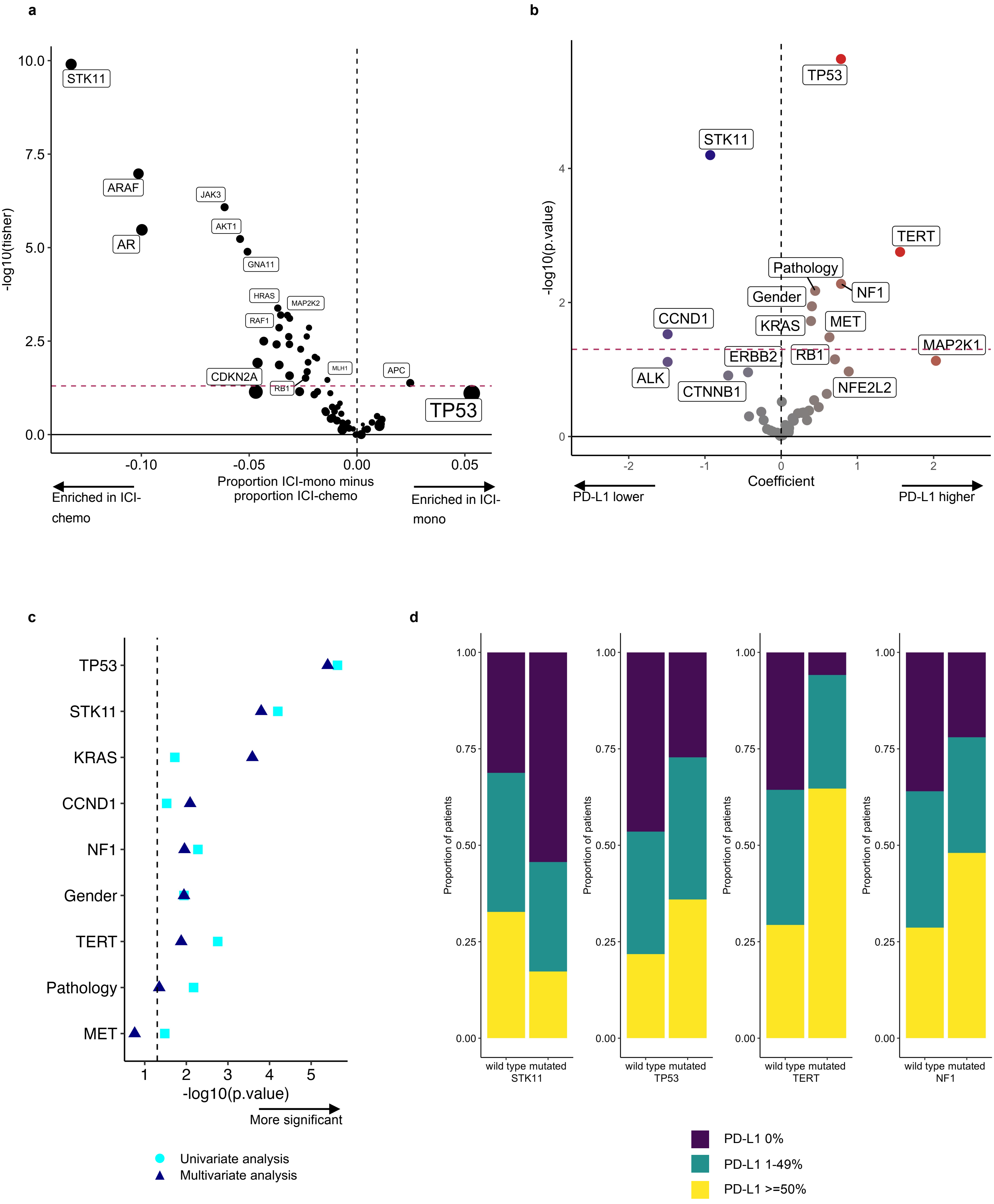
a
b
c
d
STK11
ARAF
AR
JAK3
AKT1
GNA11
HRAS
MAP2K2
RAF1
CDKN2A
RB1
MLH1
APC
TP53
10.0
7.5
5.0
2.5
0.0
-0.10
-0.05
0.00
0.05
-log10(fisher)
Proportion ICI-mono minus proportion ICI-chemo
Enriched in ICI-chemo
Enriched in ICI-mono
TP53
STK11
TERT
Pathology
NF1
Gender
CCND1
KRAS
MET
MAP2K1
ERBB2
RB1
ALK
CTNNB1
NFE2L2
-log10(p.value)
Coefficient
PD-L1 lower
PD-L1 higher
-2
-1
0
1
2
4
TP53
STK11
KRAS
CCND1
NF1
Gender
TERT
Pathology
MET
1
2
3
4
5
-log10(p.value)
More significant
Univariate analysis
Multivariate analysis
Proportion of patients
1.00
0.75
0.50
0.25
0.00
wild type mutated
STK11
TP53
TERT
NF1
PD-L1 0%
PD-L1 1-49%
PD-L1 >=50%

Supplementary Figure 4. **Association between gene alterations and treatment strategies or PD-L1 expression level in the MDACC-primary cohort (n=1,133)**. Volcano plot of an enrichment of genomic alterations associated with **a,** immune checkpoint inhibitor (ICI) monotherapy (ICI-mono) or ICI with concurrent chemotherapy (ICI-chemo) or **b,** PD-L1 expression. **c,** P-value for the association gene alterations with PD-L1 expression in uni- (light blue square) and multivariate (dark blue triangle) analysis; ordinal logistic regression using the MASS R package was performed. **d,** Distribution of PD-L1 positivity in the patients with and without alterations in *STK11*, *TP53*, *TERT* and *NF1*. Source data are provided as a Source Data file.

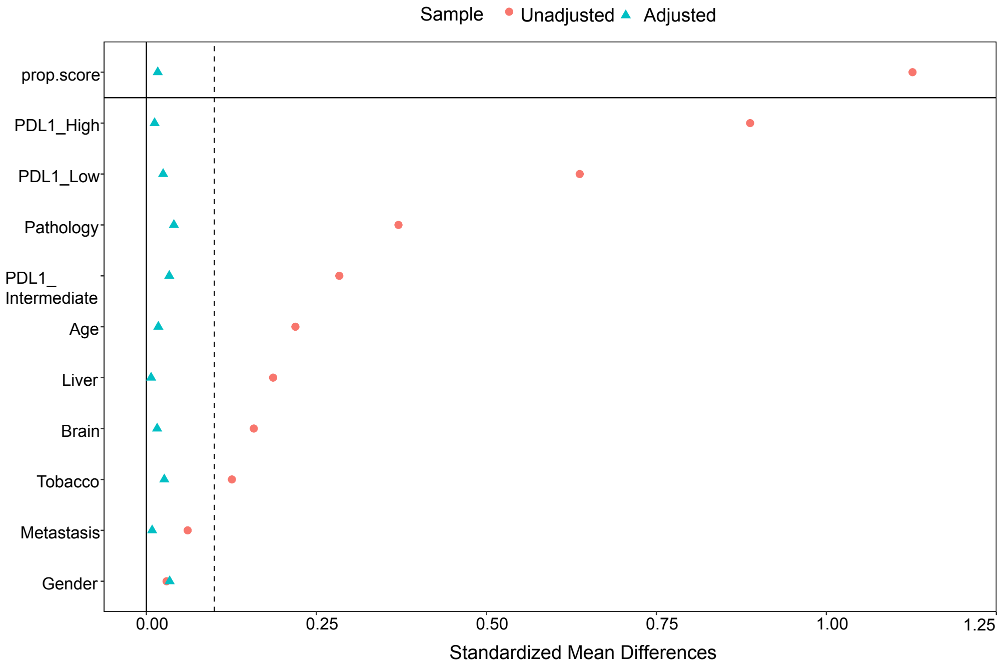
Sample
Unadjusted
Adjusted
prop.score
PDL1_High
PDL1_Low
Pathology
PDL1_
Intermediate
Age
Liver
Brain
Tobacco
Metastasis
Gender
0.00
0.25
0.50
0.75
1.00
1.25
Standardized Mean Differences

Supplementary Figure 5. **Standardized mean differences before and after propensity-score based adjustment using the inverse probability of treatment weighting (IPTW) methodology on first-line patients (n=675) with available PD-L1 expression in the MDACC-primary cohort**. Pre- (red) and post- (blue) weighting balance in covariates assessed by standardized mean difference. Source data are provided as a Source Data file.

a

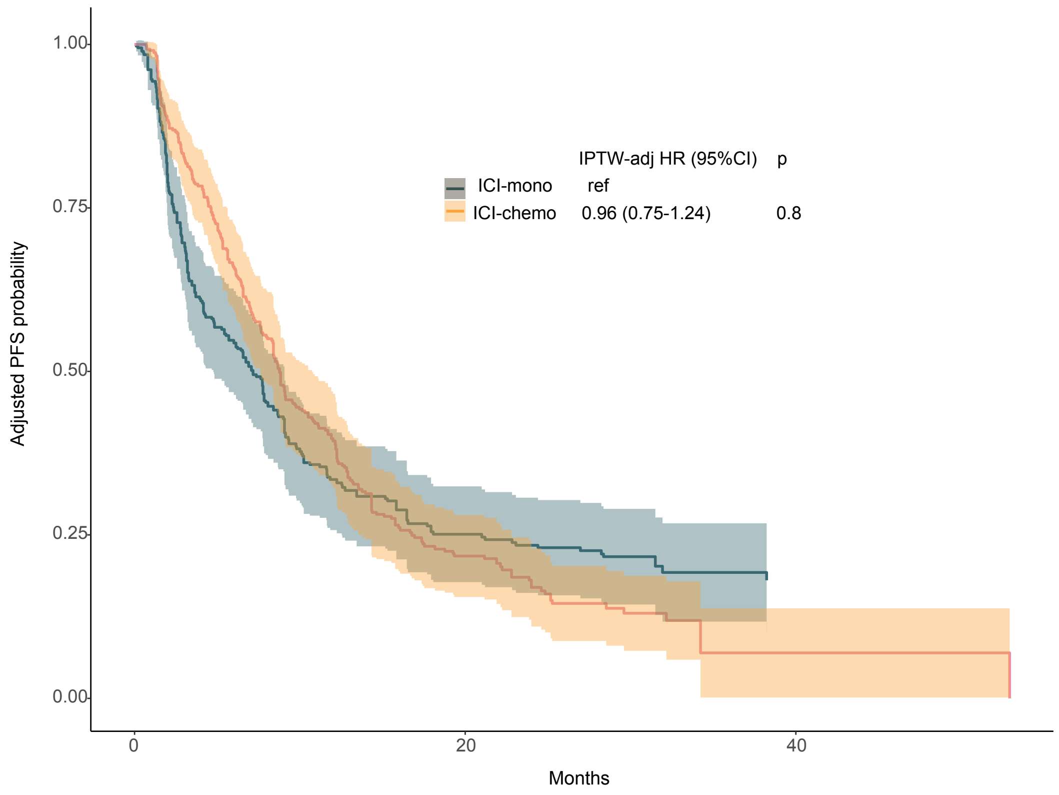
IPTW-adj HR (95%CI) p
ICI-mono ref
ICI-chemo 0.96 (0.75-1.24) 0.8
1.00
0.75
0.50
0.25
0.00
Adjusted PFS probability
0
20
40
Months

b

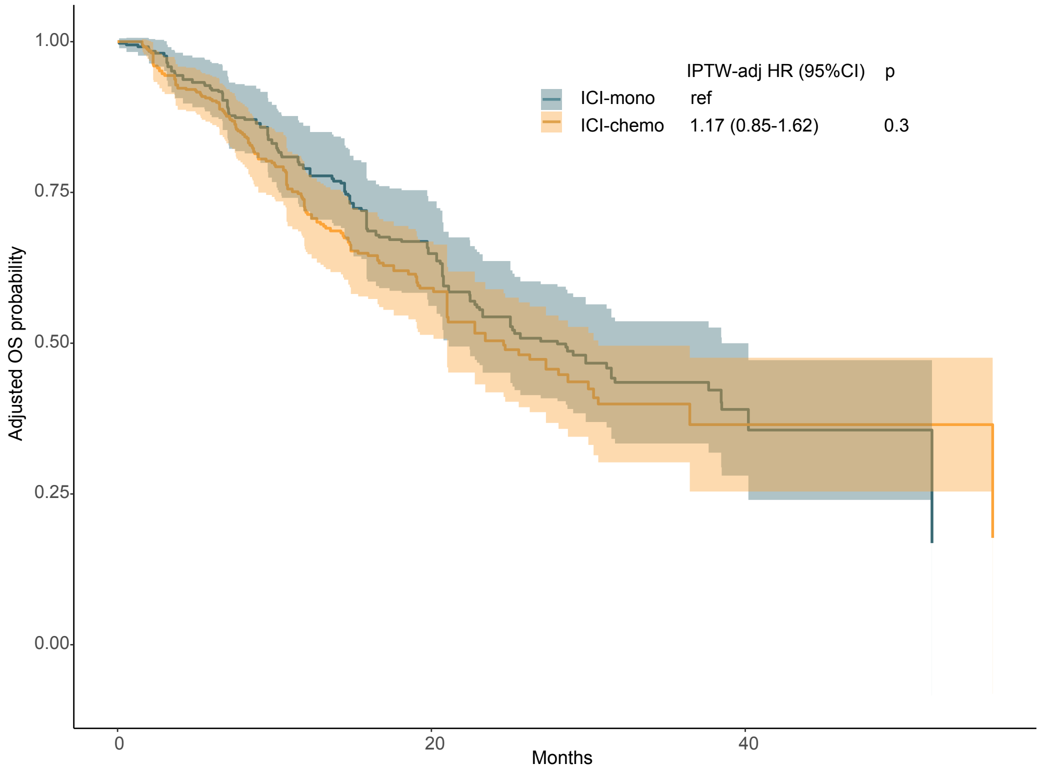
IPTW-adj HR (95%CI) p
ICI-mono ref
ICI-chemo 1.17 (0.85-1.62) 0.3
1.00
0.75
0.50
0.25
0.00
Adjusted OS probability
0
20
40
Months

Supplementary Figure 6. **Kaplan-meier plot stratified by immune checkpoint inhibitor (ICI) monotherapy (ICI-mono) vs ICI chemotherapy (ICI-chemo) in the inverse probability of treatment weighting (IPTW) adjusted MDACC-primary first-line patients with available PD-L1 expression (n=534)**. **a,** progression-free survival (PFS); **b,** overall survival (OS). 95% confidence intervals (95% CI) are reported under each of the KM estimates and as shadowed area. IPTW adjusted hazard ratio (IPTW-adj HR) and p values are reported according to Cox proportional hazards regression with IPTW-adjusted analysis. Source data are provided as a Source Data file.

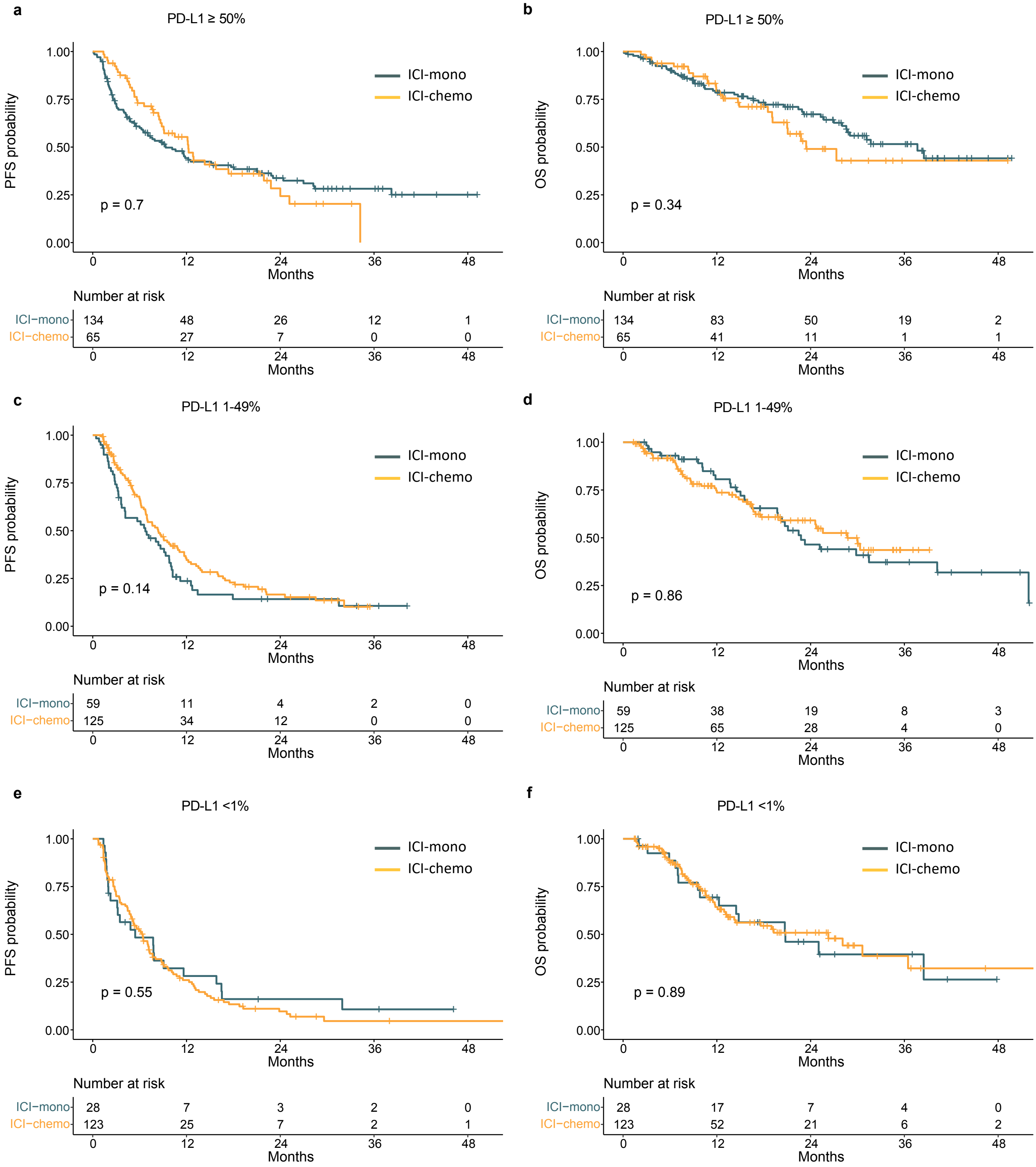
a
PD-L1 ≥ 50%
PFS probability
1.00
0.75
0.50
0.25
0.00
p = 0.7
ICI-mono
ICI-chemo
0
12
24
36
48
Months
Number at risk
ICI−mono 134 48 26 12 1
ICI−chemo 65 27 7 0 0
0
12
24
36
48
Months
b
PD-L1 ≥ 50%
OS probability
1.00
0.75
0.50
0.25
0.00
p = 0.34
ICI-mono
ICI-chemo
0
12
24
36
48
Months
Number at risk
ICI−mono 134 83 50 19 2
ICI−chemo 65 41 11 1 1
0
12
24
36
48
Months
c
PD-L1 1-49%
PFS probability
1.00
0.75
0.50
0.25
0.00
p = 0.14
ICI-mono
ICI-chemo
0
12
24
36
48
Months
Number at risk
ICI−mono 59 11 4 2 0
ICI−chemo 125 34 12 0 0
0
12
24
36
48
Months
d
PD-L1 1-49%
OS probability
1.00
0.75
0.50
0.25
0.00
p = 0.86
ICI-mono
ICI-chemo
0
12
24
36
48
Months
Number at risk
ICI−mono 59 38 19 8 3
ICI−chemo 125 65 28 4 0
0
12
24
36
48
Months
e
PD-L1 <1%
PFS probability
1.00
0.75
0.50
0.25
0.00
p = 0.55
ICI-mono
ICI-chemo
0
12
24
36
48
Months
Number at risk
ICI−mono 28 7 3 2 0
ICI−chemo 123 25 7 2 1
0
12
24
36
48
Months
f
PD-L1 <1%
OS probability
1.00
0.75
0.50
0.25
0.00
p = 0.89
ICI-mono
ICI-chemo
0
12
24
36
48
Months
Number at risk
ICI−mono 28 17 7 4 0
ICI−chemo 123 52 21 6 2
0
12
24
36
48
Months

Supplementary Figure 7. **Outcomes in patients treated with immune checkpoint inhibitor (ICI) monotherapy (ICI-mono) vs ICI chemotherapy (ICI-chemo) in the MDACC-primary first-line cohort (n=675), stratified by PD-L1 expression**. Kaplan-meier plot of **a,** progression-free survival (PFS) and **b,** overall survival (OS) in patients with PD-L1 ≥ 50%. **c,** PFS and **d,** OS in patients with PD-L1: 1-49%. **e,** PFS and **f,** OS in patients with PD-L1 < 1%. P values were calculated using log-rank analysis. Source data are provided as a Source Data file.

a

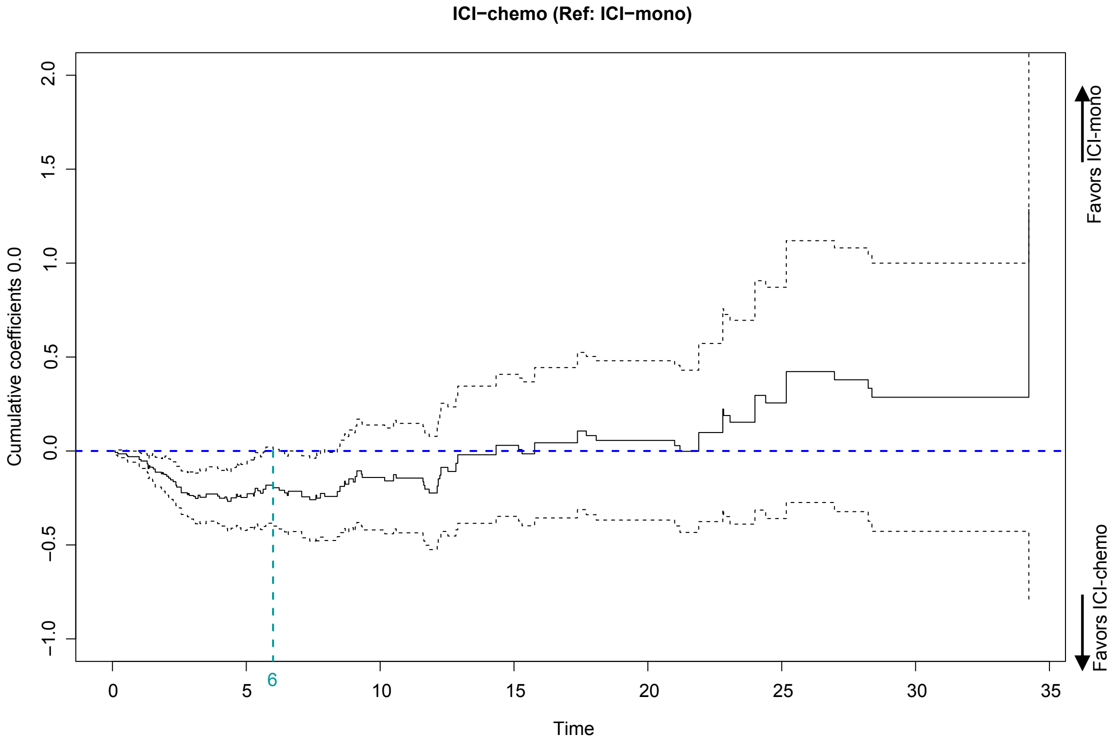

ICI−chemo (Ref: ICI−mono)
Cumulative coefficients 0.0
2.0
1.5
1.0
0.5
0.0
−0.5
−1.0
0
5
6
10
15
20
25
30
35
Time
Favors ICI-mono
Favors ICI-chemo

b

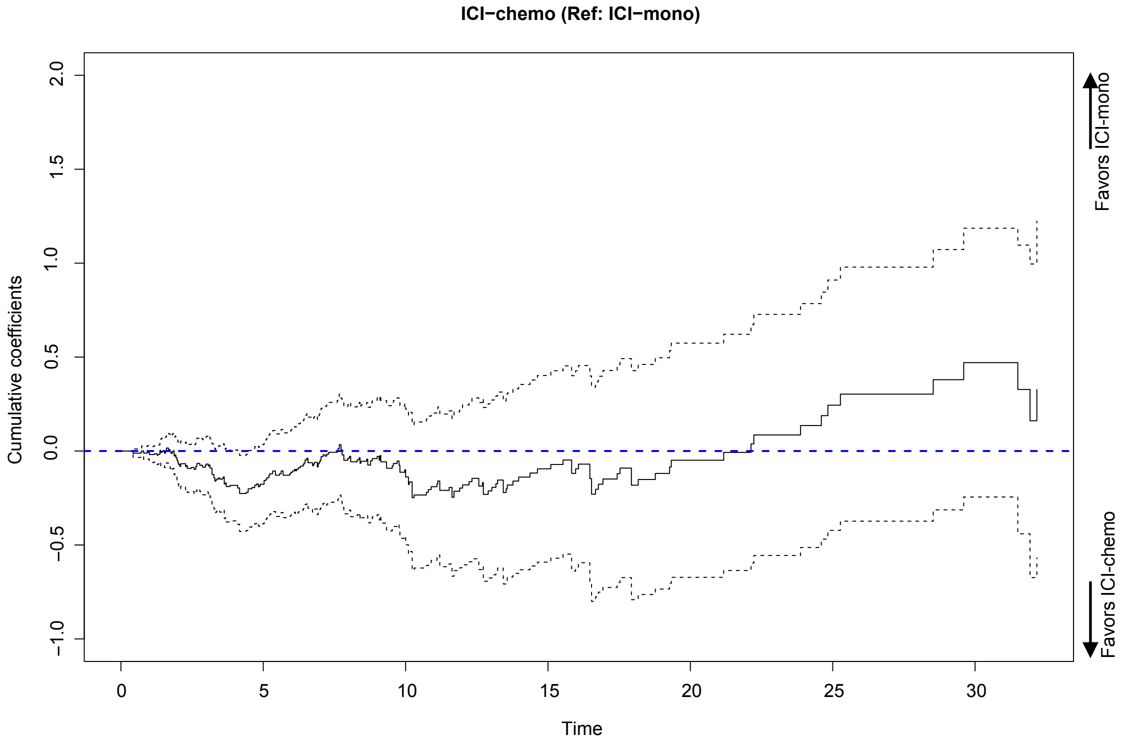

ICI−chemo (Ref: ICI−mono)
Cumulative coefficients
2.0
1.5
1.0
0.5
0.0
−0.5
−1.0
0
5
10
15
20
25
30
Time
Favors ICI-mono
Favors ICI-chemo

Supplementary Figure 8. **Aalen's additive hazard model on progression-free survival (PFS) with a 95% confidence interval for patients treated with immune checkpoint inhibitor (ICI) monotherapy (ICI-mono) vs ICI chemotherapy (ICI-chemo), stratified by PD-L1 expression**. **a,** PD-L1 ≥ 50% (n=199); **b,** PD-L1 <50% (n=335). Source data are provided as a Source Data file.

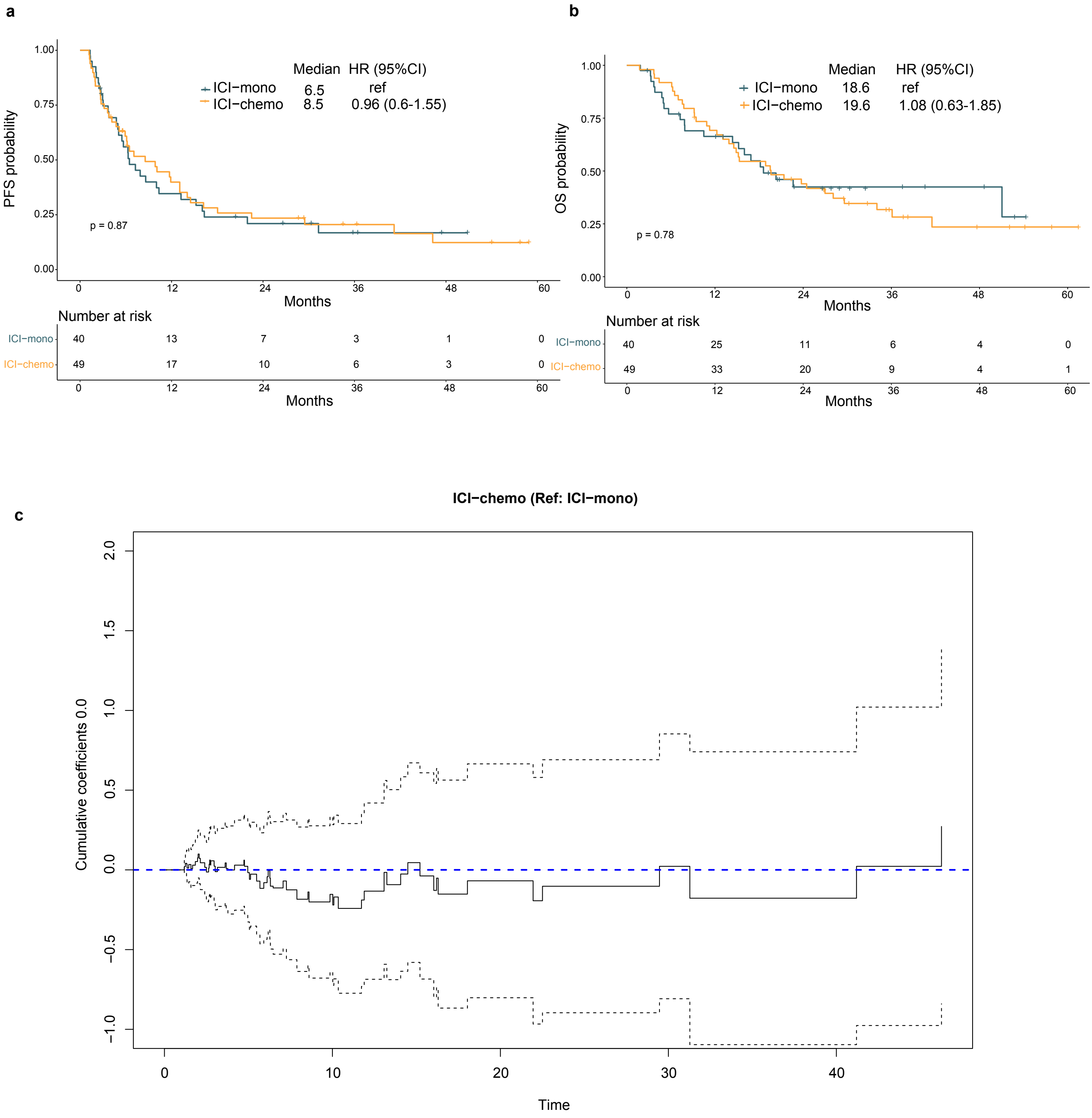
a
PFS probability
1.00
0.75
0.50
0.25
0.00
Median HR (95%CI)
ICI−mono 6.5 ref
ICI−chemo 8.5 0.96 (0.6-1.55)
p = 0.87
0 12 24 36 48 60
Months
Number at risk
ICI−mono 40 13 7 3 1 0
ICI−chemo 49 17 10 6 3 0
0 12 24 36 48 60
Months
b
OS probability
1.00
0.75
0.50
0.25
0.00
Median HR (95%CI)
ICI−mono 18.6 ref
ICI−chemo 19.6 1.08 (0.63-1.85)
p = 0.78
0 12 24 36 48 60
Months
Number at risk
ICI−mono 40 25 11 6 4 0
ICI−chemo 49 33 20 9 4 1
0 12 24 36 48 60
Months
ICI−chemo (Ref: ICI−mono)
c
Cumulative coefficients 0.0
2.0
1.5
1.0
0.5
0.0
−0.5
−1.0
0 10 20 30 40
Time

Supplementary Figure 9. **Clinical outcomes in the Mayo validation cohort**. **a,** progression-free survival (PFS) and **b,** overall survival (OS) between immune checkpoint inhibitor (ICI) monotherapy (ICI-mono) vs ICI chemotherapy (ICI-chemo). Hazard ratio (HR) with 95% confidence interval (95%CI) and p values were calculated using log-rank analysis. **c,** Aalen's additive hazard model on PFS. Coefficient < 0 favors ICI-chemo. Dashed grey lines indicate 95% confidence interval. Source data are provided as a Source Data file.

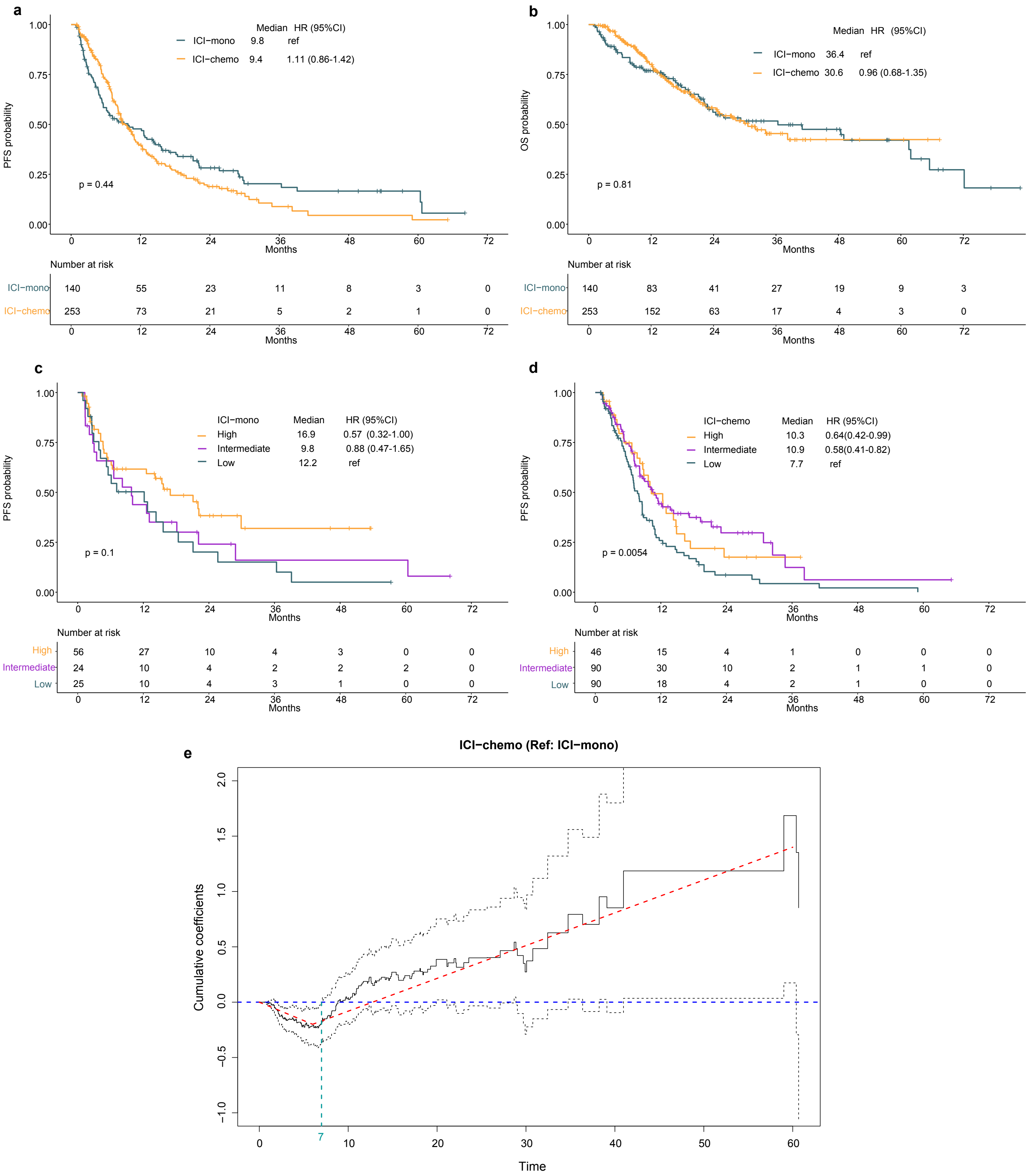
a
PFS probability
Median HR (95%CI)
ICI−mono 9.8 ref
ICI−chemo 9.4 1.11 (0.86-1.42)
p = 0.44
Months
Number at risk
ICI−mono 140 55 23 11 8 3 0
ICI−chemo 253 73 21 5 2 1 0
b
OS probability
Median HR (95%CI)
ICI−mono 36.4 ref
ICI−chemo 30.6 0.96 (0.68-1.35)
p = 0.81
Months
Number at risk
ICI−mono 140 83 41 27 19 9 3
ICI−chemo 253 152 63 17 4 3 0
c
PFS probability
ICI−mono Median HR (95%CI)
High 16.9 0.57 (0.32-1.00)
Intermediate 9.8 0.88 (0.47-1.65)
Low 12.2 ref
p = 0.1
Months
Number at risk
High 56 27 10 4 3 0 0
Intermediate 24 10 4 2 2 2 0
Low 25 10 4 3 1 0 0
d
PFS probability
ICI−chemo Median HR (95%CI)
High 10.3 0.64(0.42-0.99)
Intermediate 10.9 0.58(0.41-0.82)
Low 7.7 ref
p = 0.0054
Months
Number at risk
High 46 15 4 1 0 0 0
Intermediate 90 30 10 2 1 1 0
Low 90 18 4 2 1 0 0
e
ICI−chemo (Ref: ICI−mono)
Cumulative coefficients
Time
7

Supplementary Figure 10. **Clinical outcomes in the MDACC validation cohort**. **a,** progression-free survival (PFS) and **b,** overall survival (OS) between immune checkpoint inhibitor (ICI) monotherapy (ICI-mono) vs ICI chemotherapy (ICI-chemo). **c,** PFS in ICI-mono stratified by PD-L1. **d,** PFS in ICI-chemo stratified by PD-L1. Hazard ratio (HR) with 95% confidence interval (95%CI) and p values were calculated using log-rank analysis. **e,** Aalen's additive hazard model on PFS. Coefficient < 0 favors ICI-chemo. Dashed grey lines indicate 95% confidence interval. Source data are provided as a Source Data file.

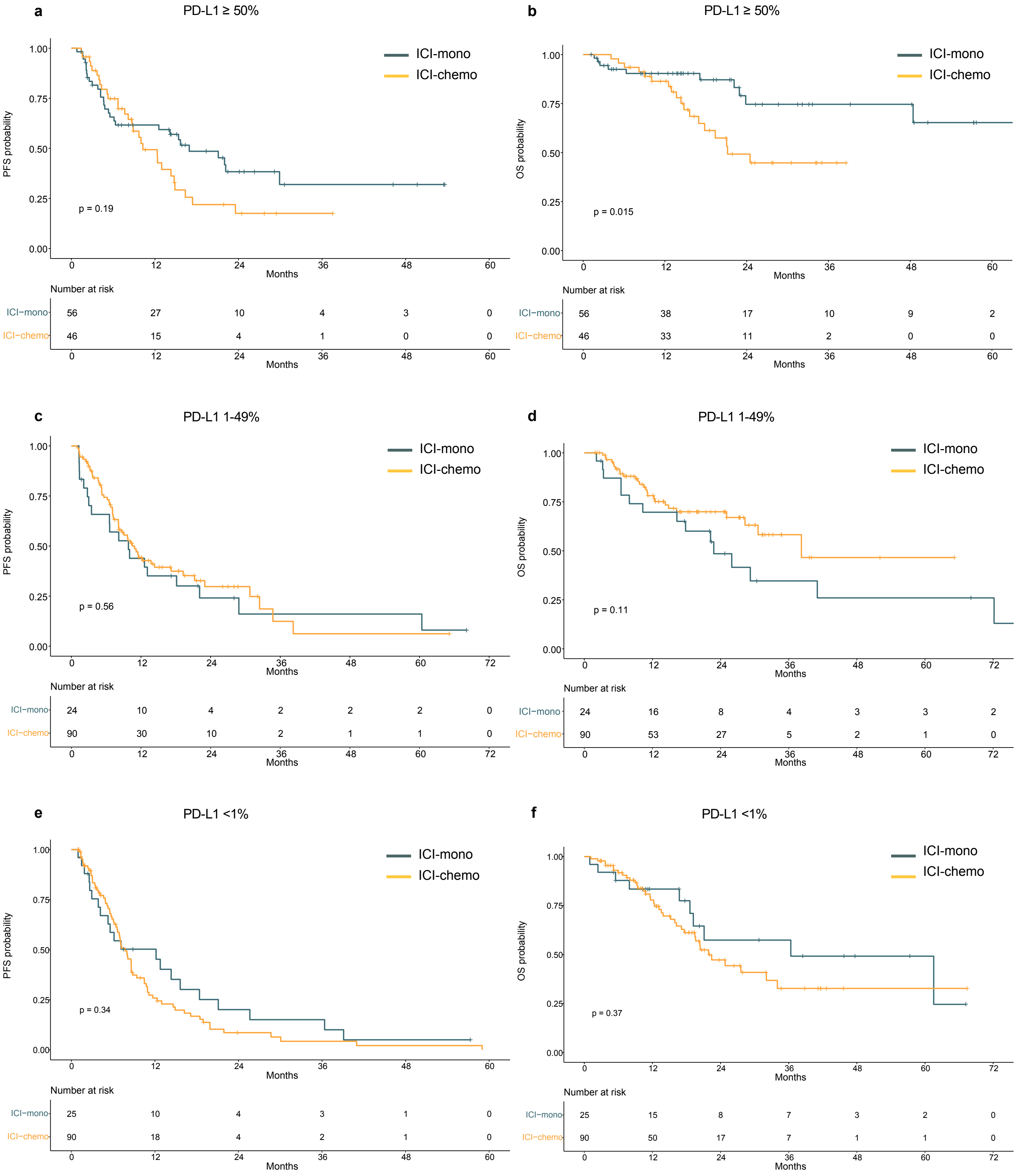
a
PD-L1 ≥ 50%
ICI-mono
ICI-chemo
PFS probability
p = 0.19
Months
Number at risk
ICI−mono 56 27 10 4 3 0
ICI−chemo 46 15 4 1 0 0
b
PD-L1 ≥ 50%
ICI-mono
ICI-chemo
OS probability
p = 0.015
Months
Number at risk
ICI−mono 56 38 17 10 9 2
ICI−chemo 46 33 11 2 0 0
c
PD-L1 1-49%
ICI-mono
ICI-chemo
PFS probability
p = 0.56
Months
Number at risk
ICI−mono 24 10 4 2 2 2 0
ICI−chemo 90 30 10 2 1 1 0
d
PD-L1 1-49%
ICI-mono
ICI-chemo
OS probability
p = 0.11
Months
Number at risk
ICI−mono 24 16 8 4 3 3 2
ICI−chemo 90 53 27 5 2 1 0
e
PD-L1 <1%
ICI-mono
ICI-chemo
PFS probability
p = 0.34
Months
Number at risk
ICI−mono 25 10 4 3 1 0
ICI−chemo 90 18 4 2 1 0
f
PD-L1 <1%
ICI-mono
ICI-chemo
OS probability
p = 0.37
Months
Number at risk
ICI−mono 25 15 8 7 3 2 0
ICI−chemo 90 50 17 7 1 1 0

Supplementary Figure 11. **Outcomes in patients treated with immune checkpoint inhibitor (ICI) monotherapy (ICI-mono) vs ICI chemotherapy (ICI-chemo) in the MDACC-validation cohort**. Kaplan-meier plot of **a,** progression-free survival (PFS) and **b,** overall survival (OS) in patients with PD-L1 ≥ 50%. **c,** PFS and **d,** OS in patients with PD-L1: 1-49%. **e,** PFS and **f,** OS in patients with PD-L1 < 1%. P values were calculated using log-rank analysis. Source data are provided as a Source Data file.

a

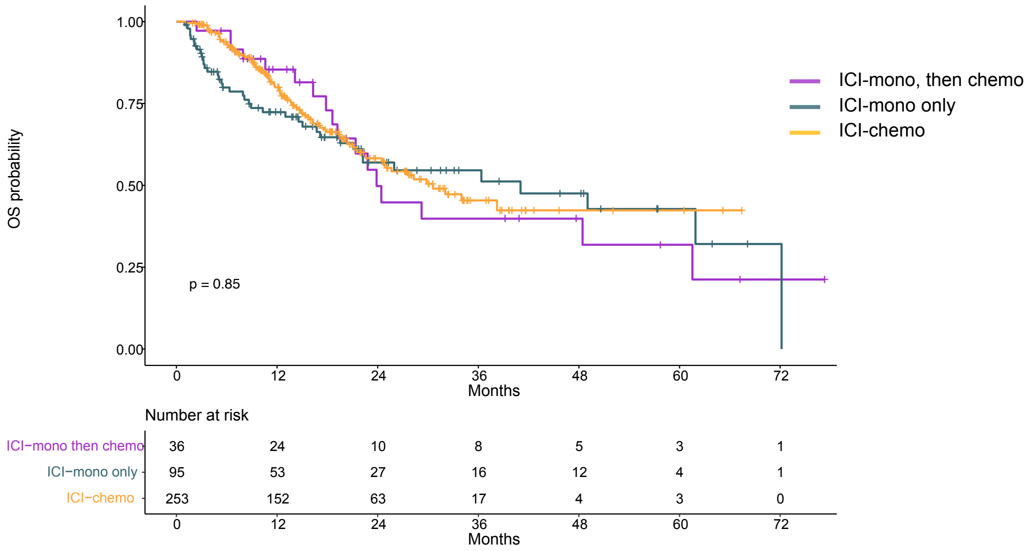
OS probability
1.00
0.75
0.50
0.25
0.00
p = 0.85
0 12 24 36 48 60 72
Months
ICI-mono, then chemo
ICI-mono only
ICI-chemo
Number at risk
ICI−mono then chemo 36 24 10 8 5 3 1
ICI−mono only 95 53 27 16 12 4 1
ICI−chemo 253 152 63 17 4 3 0
0 12 24 36 48 60 72
Months

b

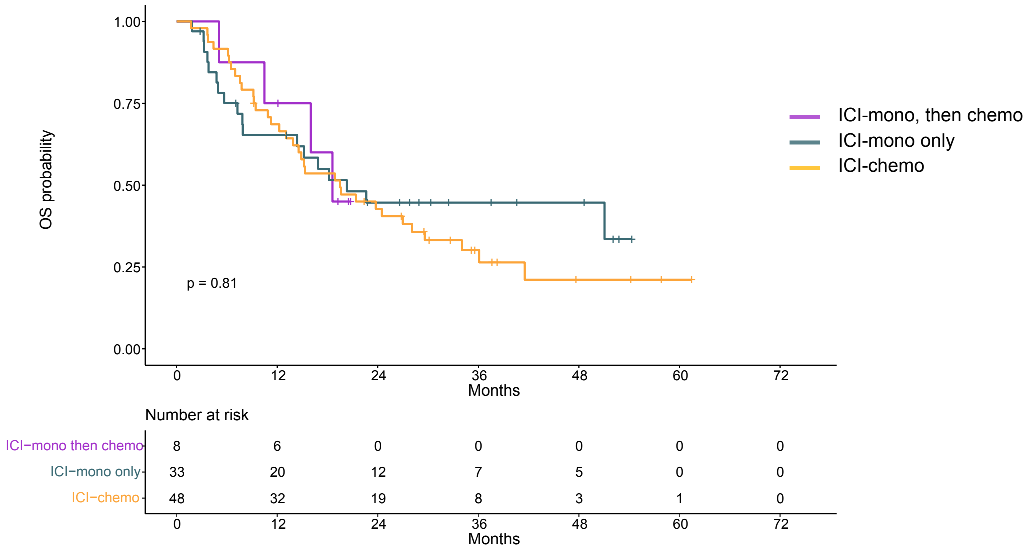
OS probability
1.00
0.75
0.50
0.25
0.00
p = 0.81
0 12 24 36 48 60 72
Months
ICI-mono, then chemo
ICI-mono only
ICI-chemo
Number at risk
ICI−mono then chemo 8 6 0 0 0 0 0
ICI−mono only 33 20 12 7 5 0 0
ICI−chemo 48 32 19 8 3 1 0
0 12 24 36 48 60 72
Months

c

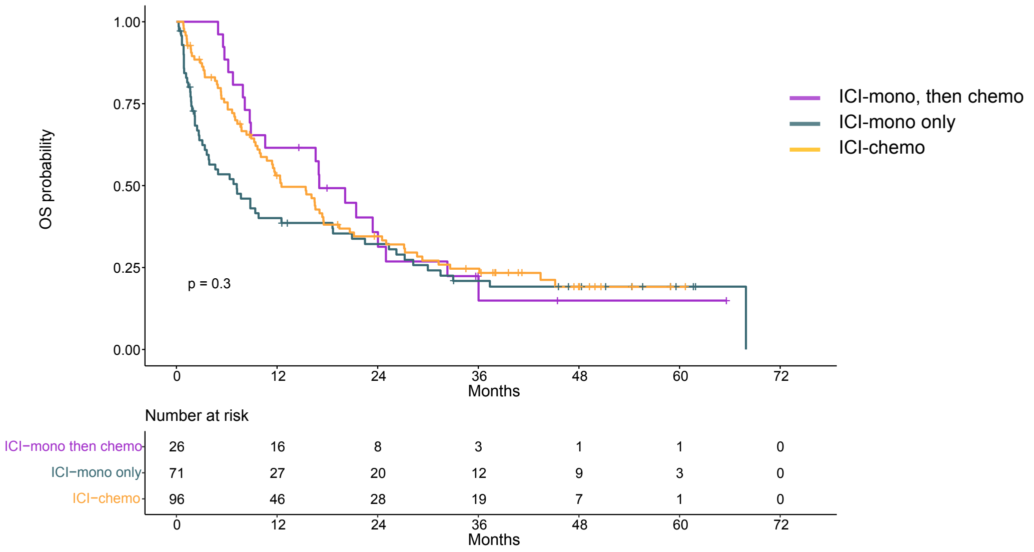
OS probability
1.00
0.75
0.50
0.25
0.00
p = 0.3
0 12 24 36 48 60 72
Months
ICI-mono, then chemo
ICI-mono only
ICI-chemo
Number at risk
ICI−mono then chemo 26 16 8 3 1 1 0
ICI−mono only 71 27 20 12 9 3 0
ICI−chemo 96 46 28 19 7 1 0
0 12 24 36 48 60 72
Months

Supplementary Figure 12. **Analyses of overall survival (OS) stratified by 2nd-line therapy in the validation cohorts**. Groups definition: immune checkpoint inhibitor monotherapy (ICI-mono) followed by chemotherapy (ICI-mono then chemo); ICI-mono only; ICI with concurrent chemotherapy (ICI-chemo). **a,** MDACC validation cohort; **b,** Mayo cohort; and **c,** MGH cohort. P values were calculated using log-rank analysis. Source data are provided as a Source Data file.

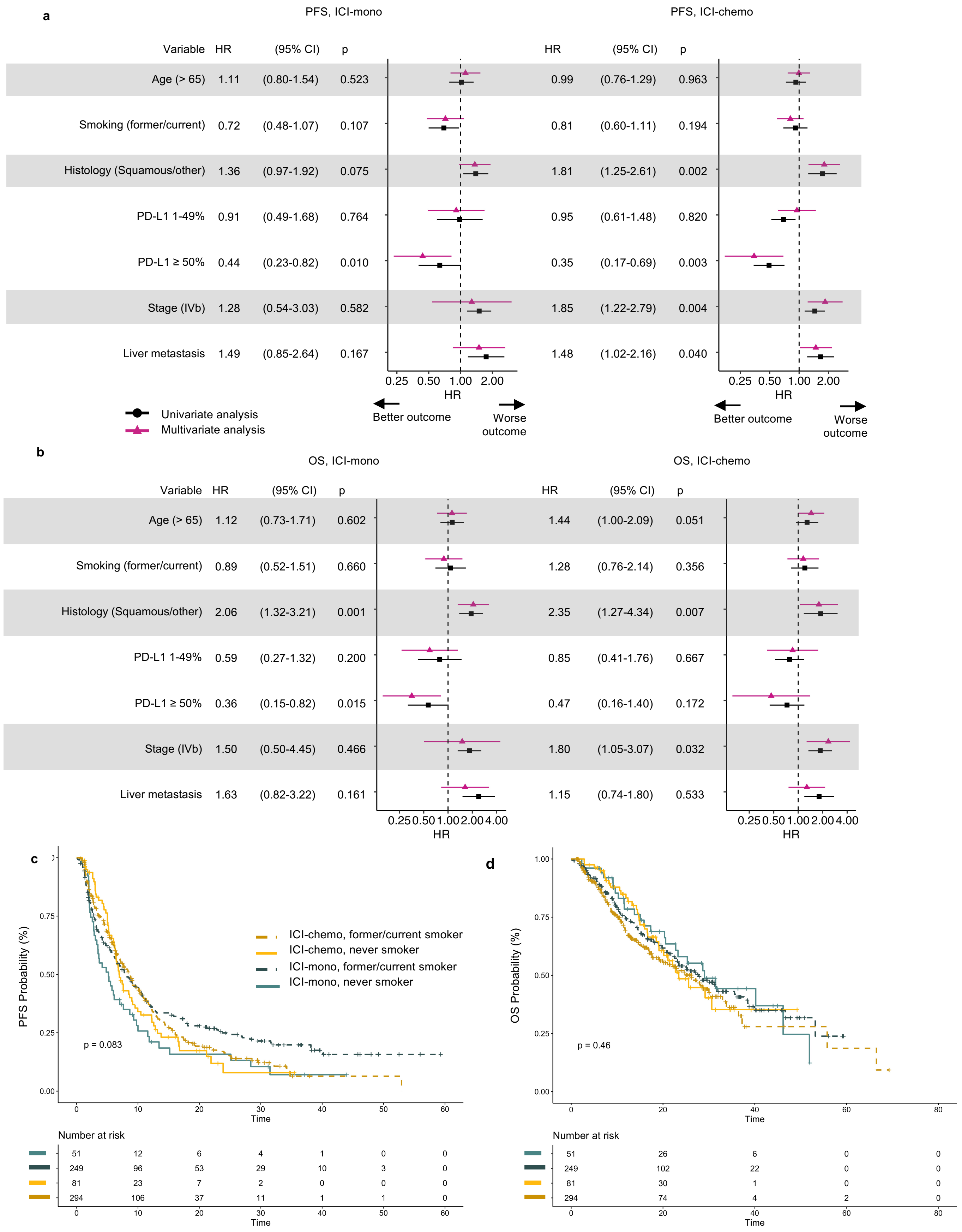
a
PFS, ICI-mono
PFS, ICI-chemo
Variable HR (95% CI) p
Age (> 65) 1.11 (0.80-1.54) 0.523 | 0.99 (0.76-1.29) 0.963
Smoking (former/current) 0.72 (0.48-1.07) 0.107 | 0.81 (0.60-1.11) 0.194
Histology (Squamous/other) 1.36 (0.97-1.92) 0.075 | 1.81 (1.25-2.61) 0.002
PD-L1 1-49% 0.91 (0.49-1.68) 0.764 | 0.95 (0.61-1.48) 0.820
PD-L1 ≥ 50% 0.44 (0.23-0.82) 0.010 | 0.35 (0.17-0.69) 0.003
Stage (IVb) 1.28 (0.54-3.03) 0.582 | 1.85 (1.22-2.79) 0.004
Liver metastasis 1.49 (0.85-2.64) 0.167 | 1.48 (1.02-2.16) 0.040
0.25 0.50 1.00 2.00
HR
Better outcome
Worse outcome
Univariate analysis
Multivariate analysis
b
OS, ICI-mono
OS, ICI-chemo
Variable HR (95% CI) p
Age (> 65) 1.12 (0.73-1.71) 0.602 | 1.44 (1.00-2.09) 0.051
Smoking (former/current) 0.89 (0.52-1.51) 0.660 | 1.28 (0.76-2.14) 0.356
Histology (Squamous/other) 2.06 (1.32-3.21) 0.001 | 2.35 (1.27-4.34) 0.007
PD-L1 1-49% 0.59 (0.27-1.32) 0.200 | 0.85 (0.41-1.76) 0.667
PD-L1 ≥ 50% 0.36 (0.15-0.82) 0.015 | 0.47 (0.16-1.40) 0.172
Stage (IVb) 1.50 (0.50-4.45) 0.466 | 1.80 (1.05-3.07) 0.032
Liver metastasis 1.63 (0.82-3.22) 0.161 | 1.15 (0.74-1.80) 0.533
0.25 0.50 1.00 2.00 4.00
HR
c
PFS Probability (%)
0.75
0.50
0.25
0.00
p = 0.083
ICI-chemo, former/current smoker
ICI-chemo, never smoker
ICI-mono, former/current smoker
ICI-mono, never smoker
0 10 20 30 40 50 60
Time
Number at risk
51 12 6 4 1 0 0
249 96 53 29 10 3 0
81 23 7 2 0 0 0
294 106 37 11 1 1 0
0 10 20 30 40 50 60
Time
d
OS Probability (%)
1.00
0.75
0.50
0.25
0.00
p = 0.46
0 20 40 60 80
Time
Number at risk
51 26 6 0 0
249 102 22 0 0
81 30 1 0 0
294 74 4 2 0
0 20 40 60 80
Time

Supplementary Figure 13. **Clinicopathological predictors of outcome by treatment strategy in patients treated in the first-line setting (MDACC-primary, 1st line; n=675)**. Forest plot of clinical variables associated with **a,** progression-free survival (PFS) and **b,** overall-survival (OS). Immune checkpoint inhibitor (ICI) monotherapy (ICI-mono) left panel, ICI with chemotherapy (ICI-chemo) right panel. Data are presented as the hazard ratio with error bars showing 95% confidence interval; values from univariate analysis in black, multivariate in pink. Cox proportional hazards regression models were applied to calculate the hazard ratio and p values. Kaplan-meier plot by smoking status: **c,** PFS; **d,** OS; p values were calculated using log-rank analysis. Source data are provided as a Source Data file.

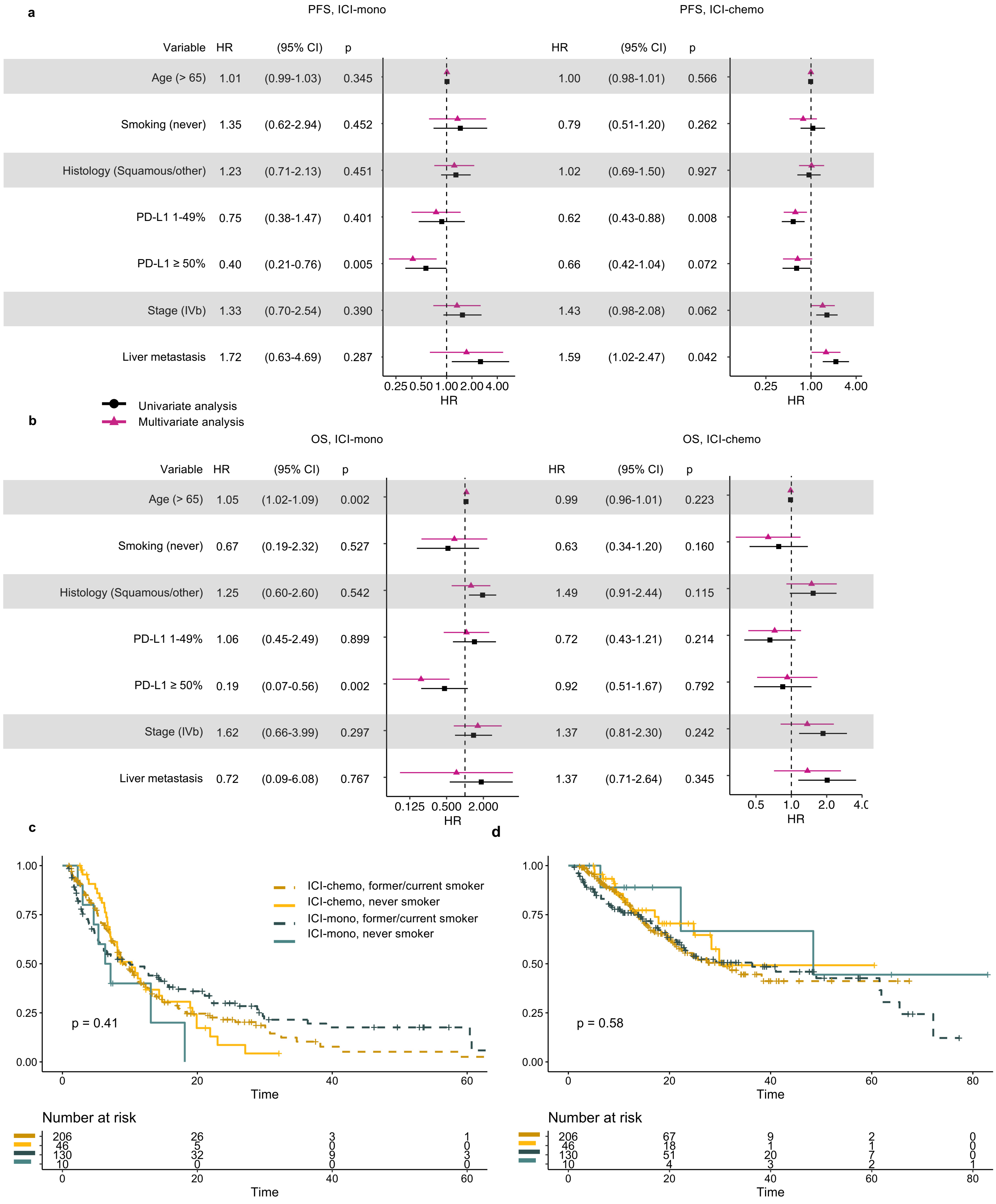
a
PFS, ICI-mono
PFS, ICI-chemo
Variable HR (95% CI) p
Age (> 65) 1.01 (0.99-1.03) 0.345
Smoking (never) 1.35 (0.62-2.94) 0.452
Histology (Squamous/other) 1.23 (0.71-2.13) 0.451
PD-L1 1-49% 0.75 (0.38-1.47) 0.401
PD-L1 ≥ 50% 0.40 (0.21-0.76) 0.005
Stage (IVb) 1.33 (0.70-2.54) 0.390
Liver metastasis 1.72 (0.63-4.69) 0.287
0.25 0.50 1.00 2.00 4.00
HR
HR (95% CI) p
1.00 (0.98-1.01) 0.566
0.79 (0.51-1.20) 0.262
1.02 (0.69-1.50) 0.927
0.62 (0.43-0.88) 0.008
0.66 (0.42-1.04) 0.072
1.43 (0.98-2.08) 0.062
1.59 (1.02-2.47) 0.042
0.25 1.00 4.00
HR
Univariate analysis
Multivariate analysis
b
OS, ICI-mono
OS, ICI-chemo
Variable HR (95% CI) p
Age (> 65) 1.05 (1.02-1.09) 0.002
Smoking (never) 0.67 (0.19-2.32) 0.527
Histology (Squamous/other) 1.25 (0.60-2.60) 0.542
PD-L1 1-49% 1.06 (0.45-2.49) 0.899
PD-L1 ≥ 50% 0.19 (0.07-0.56) 0.002
Stage (IVb) 1.62 (0.66-3.99) 0.297
Liver metastasis 0.72 (0.09-6.08) 0.767
0.125 0.500 2.000
HR
HR (95% CI) p
0.99 (0.96-1.01) 0.223
0.63 (0.34-1.20) 0.160
1.49 (0.91-2.44) 0.115
0.72 (0.43-1.21) 0.214
0.92 (0.51-1.67) 0.792
1.37 (0.81-2.30) 0.242
1.37 (0.71-2.64) 0.345
0.5 1.0 2.0 4.0
HR
c
1.00
0.75
0.50
0.25
0.00
ICI-chemo, former/current smoker
ICI-chemo, never smoker
ICI-mono, former/current smoker
ICI-mono, never smoker
p = 0.41
0 20 40 60
Time
Number at risk
206 26 3 1
46 5 0 0
130 32 9 3
10 0 0 0
0 20 40 60
Time
d
1.00
0.75
0.50
0.25
0.00
p = 0.58
0 20 40 60 80
Time
Number at risk
206 67 9 2 0
46 18 1 1 0
130 51 20 7 0
10 4 3 2 1
0 20 40 60 80
Time

Supplementary Figure 14. **Clinicopathological predictors of outcome by treatment strategy in the MDACC validation cohort (n=393)**. Forest plot of variables and association with **a,** progression-free survival (PFS) and **b,** overall-survival (OS). Immune checkpoint inhibitor (ICI) monotherapy (ICI-mono) left panel, ICI with chemotherapy (ICI-chemo) right panel. Data are presented as the hazard ratio with error bars showing 95% confidence interval; values from univariate analysis in black, multivariate in pink. Cox proportional hazards regression models were applied to calculate the hazard ratio and p values. Kaplan-meier plot by smoking status: **c,** PFS; **d,** OS; p values were calculated using log-rank analysis. Source data are provided as a Source Data file.

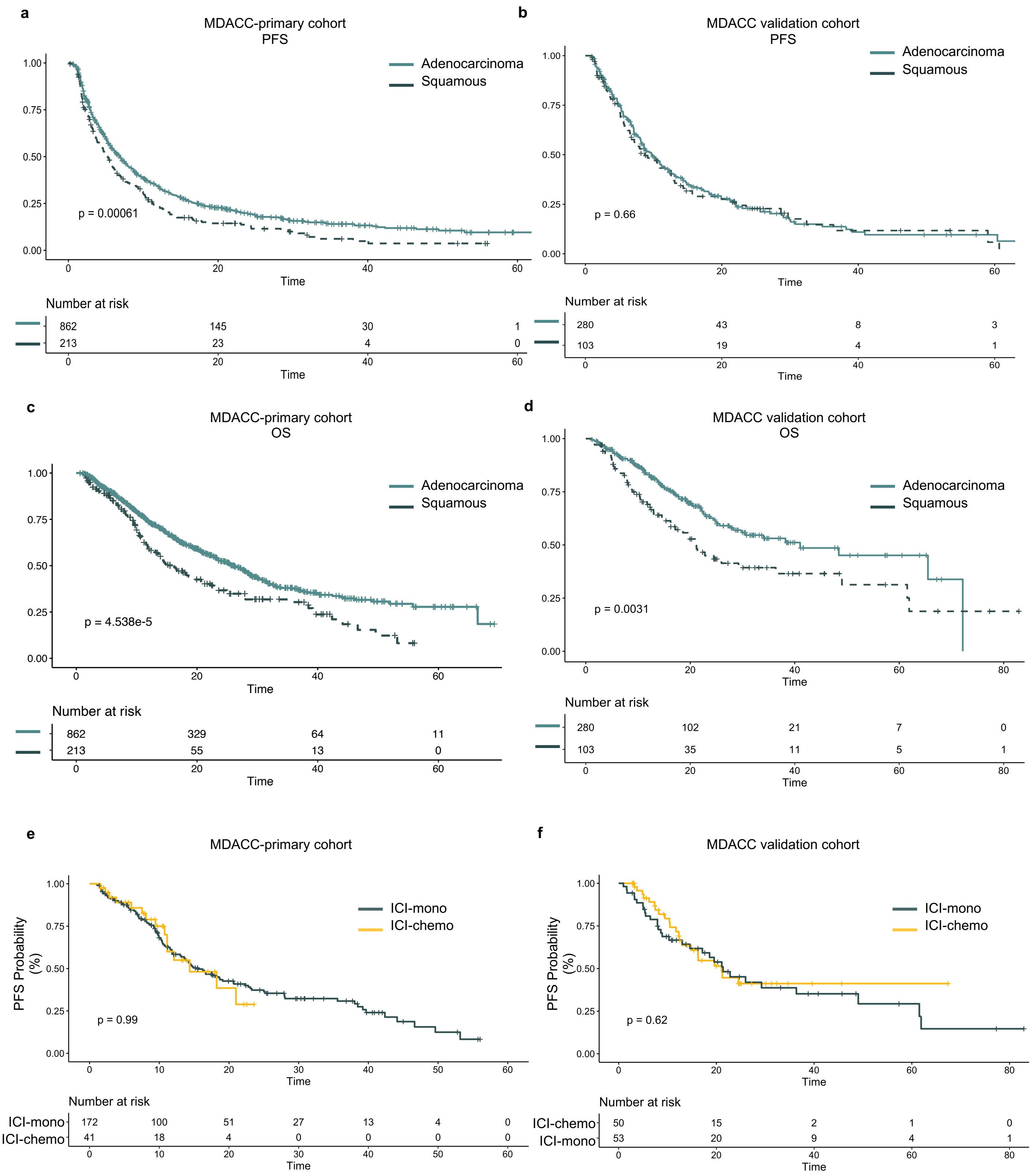

a
MDACC-primary cohort
PFS
Adenocarcinoma
Squamous
p = 0.00061
Time
Number at risk
862 145 30 1
213 23 4 0
b
MDACC validation cohort
PFS
Adenocarcinoma
Squamous
p = 0.66
Time
Number at risk
280 43 8 3
103 19 4 1
c
MDACC-primary cohort
OS
Adenocarcinoma
Squamous
p = 4.538e-5
Time
Number at risk
862 329 64 11
213 55 13 0
d
MDACC validation cohort
OS
Adenocarcinoma
Squamous
p = 0.0031
Time
Number at risk
280 102 21 7 0
103 35 11 5 1
e
MDACC-primary cohort
PFS Probability (%)
ICI-mono
ICI-chemo
p = 0.99
Time
Number at risk
ICI-mono 172 100 51 27 13 4 0
ICI-chemo 41 18 4 0 0 0 0
f
MDACC validation cohort
PFS Probability (%)
ICI-mono
ICI-chemo
p = 0.62
Time
Number at risk
ICI-chemo 50 15 2 1 0
ICI-mono 53 20 9 4 1

Supplementary Figure 15. **Association between histology and outcome**. **a,** progression-free survival (PFS) in the MDACC-primary and **b,** MDACC-validation cohorts; **c,** overall survival (OS) in the MDACC primary and **d,** MDACC validation cohort, comparing adenocarcinoma (LUAD) vs squamous cell carcinoma (LUSC). Kaplan-meier curve comparing Immune checkpoint inhibitor (ICI) monotherapy (ICI-mono) and ICI with chemotherapy (ICI-chemo) in LUSC patients in **e,** the MDACC-primary cohort and **f,** the MDACC-validation cohort. P values were calculated using log-rank analysis. Source data are provided as a Source Data file.

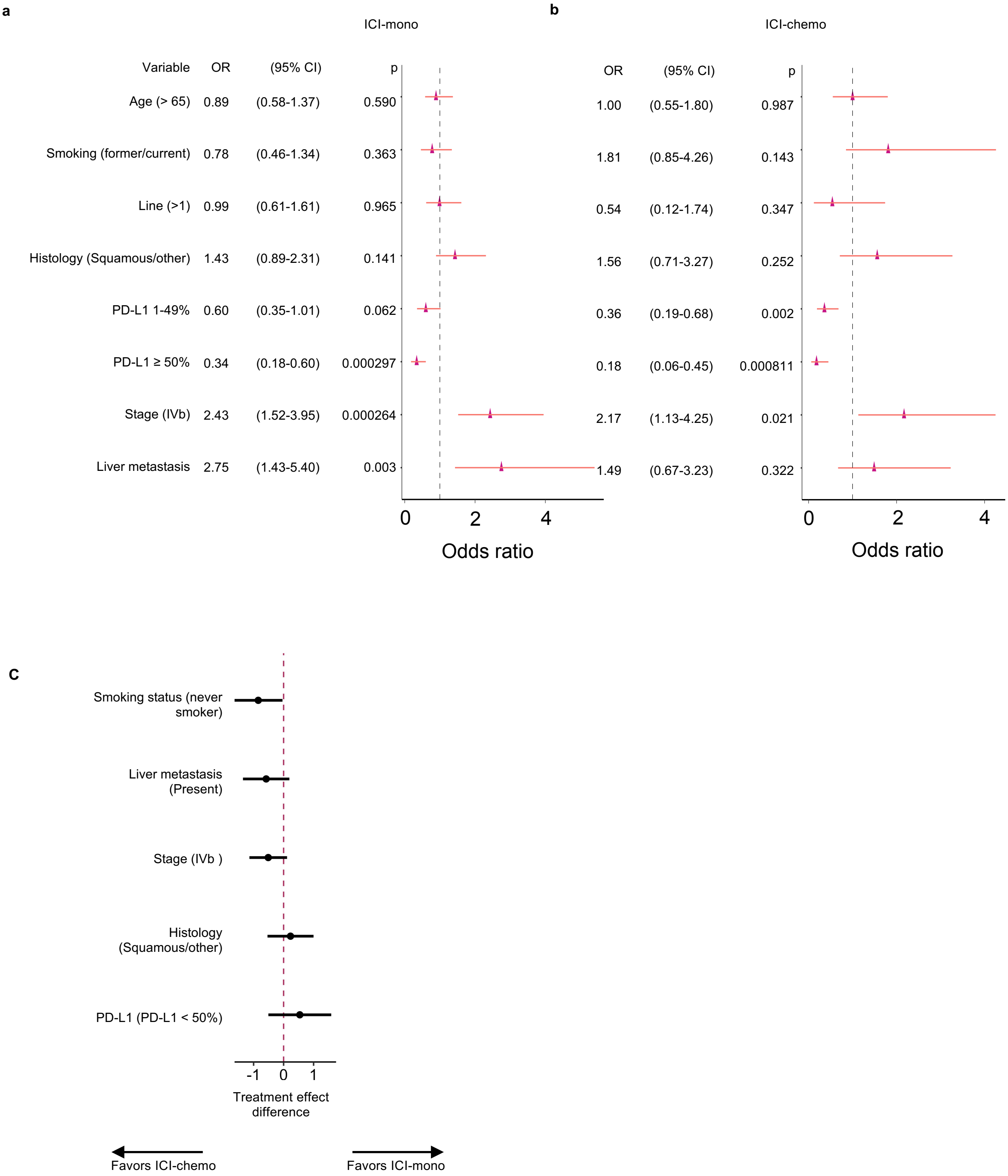
a
ICI-mono
Variable OR (95% CI) p
Age (> 65) 0.89 (0.58-1.37) 0.590
Smoking (former/current) 0.78 (0.46-1.34) 0.363
Line (>1) 0.99 (0.61-1.61) 0.965
Histology (Squamous/other) 1.43 (0.89-2.31) 0.141
PD-L1 1-49% 0.60 (0.35-1.01) 0.062
PD-L1 ≥ 50% 0.34 (0.18-0.60) 0.000297
Stage (IVb) 2.43 (1.52-3.95) 0.000264
Liver metastasis 2.75 (1.43-5.40) 0.003
0
2
4
Odds ratio
b
ICI-chemo
OR (95% CI) p
1.00 (0.55-1.80) 0.987
1.81 (0.85-4.26) 0.143
0.54 (0.12-1.74) 0.347
1.56 (0.71-3.27) 0.252
0.36 (0.19-0.68) 0.002
0.18 (0.06-0.45) 0.000811
2.17 (1.13-4.25) 0.021
1.49 (0.67-3.23) 0.322
0
2
4
Odds ratio
C
Smoking status (never smoker)
Liver metastasis (Present)
Stage (IVb)
Histology (Squamous/other)
PD-L1 (PD-L1 < 50%)
-1
0
1
Treatment effect difference
Favors ICI-chemo
Favors ICI-mono

Supplementary Figure 16. **Clinicopathological predictors of 3-month progression by treatment strategy in the MDACC primary cohort (n=1,133)**. **a,** immune checkpoint inhibitor (ICI) monotherapy (ICI-mono); **b,** ICI with chemotherapy (ICI-chemo); data are presented as the odds ratio with error bars showing 95% confidence interval; logistic regression models with adjusted effects were applied to calculate the odds ratio and p values. **c,** treatment effect analysis; data are presented as the treatment effect estimates with error bars showing the 95% confidence interval; subtee R package was used to generate treatment effect estimates. Source data are provided as a Source Data file.

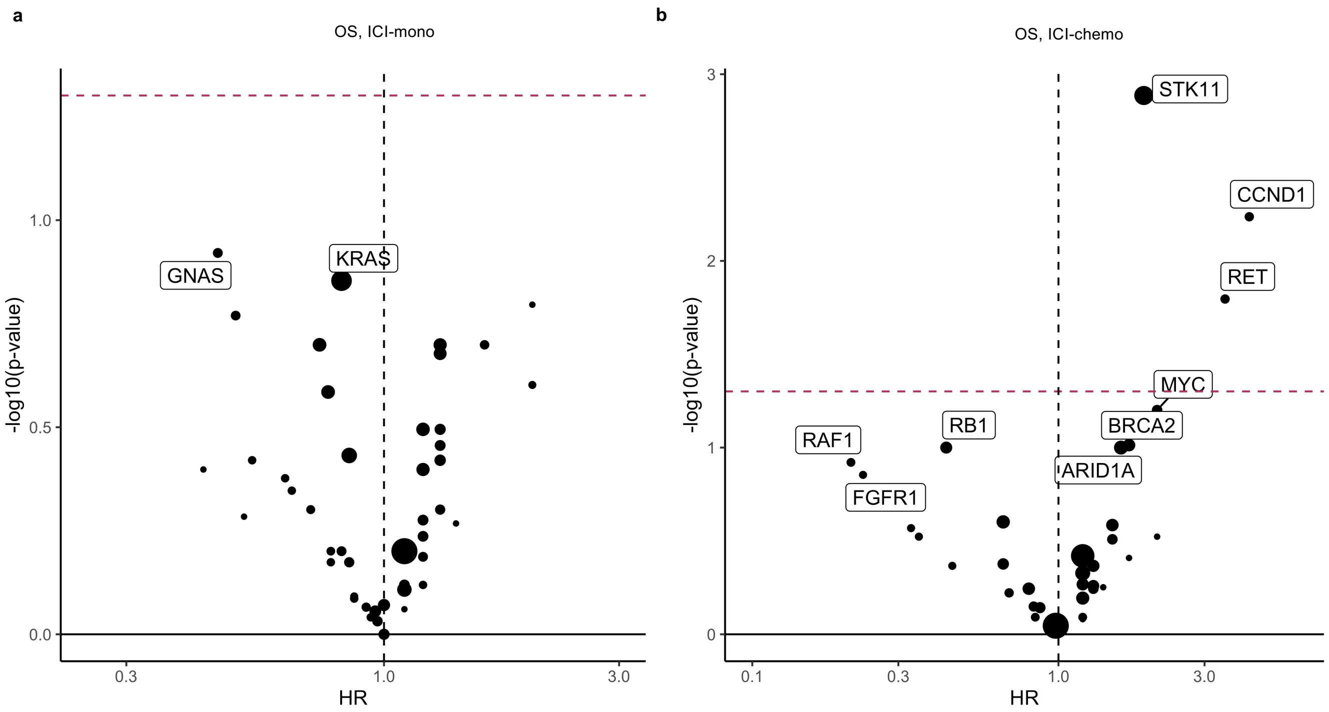

a
OS, ICI-mono
-log10(p-value)
0.0
0.5
1.0
GNAS
KRAS
0.3
1.0
3.0
HR
b
OS, ICI-chemo
-log10(p-value)
0
1
2
3
STK11
CCND1
RET
MYC
BRCA2
RB1
RAF1
ARID1A
FGFR1
0.1
0.3
1.0
3.0
HR

Supplementary Figure 17. **Association between gene alterations and overall survival (OS) in the MDACC primary cohort**. Volcano plot from univariate cox regression depicting hazard ratio (HR, x-axis) versus -log 10 (P value) (y-axis) for OS in patients treated immune checkpoint inhibitors (ICI) as **a,** monotherapy (ICI-mono) or **b,** with chemotherapy (ICI-chemo). Cox proportional hazards regression models with unadjusted effects were applied to calculate the hazard ratio and p values. Source data are provided as a Source Data file.

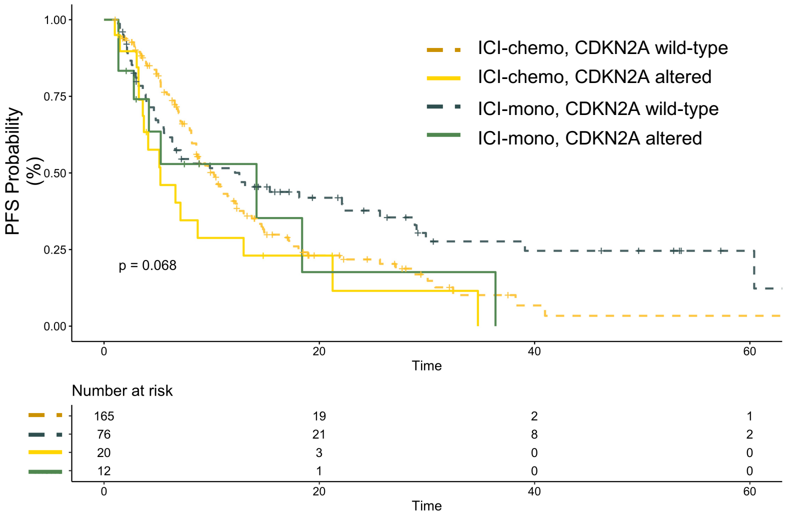

CDKN2Aalt/ICI-mono	0.789 (0.338-1.846)	0.5851
CDKN2Awt/ICI-chemo	0.640 (0.379-1.081)	0.0949
CDKN2Awt/ICI-mono	0.484 (0.274-0.857)	0.0127*

*p < 0.05

Supplementary Figure 18**. Association between *CDKN2A* alterations and progression-free survival (PFS) in MDACC-validation cohort treated with immune checkpoint inhibitor (ICI) monotherapy (ICI-mono) vs concurrent ICI-chemotherapy (ICI-chemo)**. Hazard ratio (HR) and p values within the tables were calculated using unadjusted cox proportional hazards regression models; p values in the survival plot were calculated using log-rank analysis. Source data are provided as a Source Data file.

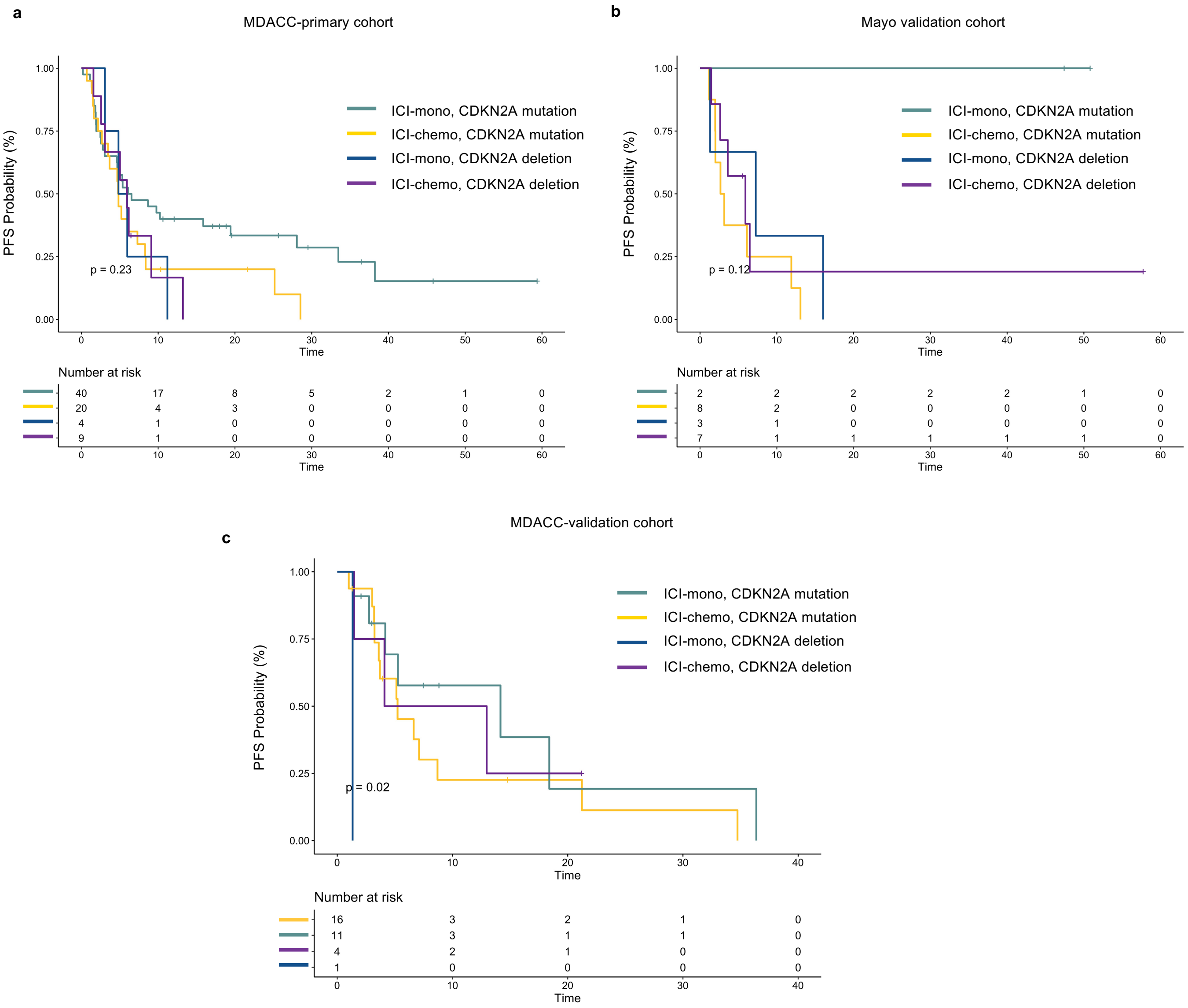

a
MDACC-primary cohort
PFS Probability (%)
1.00
0.75
0.50
0.25
0.00
p = 0.23
0
10
20
30
40
50
60
Time
ICI-mono, CDKN2A mutation
ICI-chemo, CDKN2A mutation
ICI-mono, CDKN2A deletion
ICI-chemo, CDKN2A deletion
Number at risk
40 17 8 5 2 1 0
20 4 3 0 0 0 0
4 1 0 0 0 0 0
9 1 0 0 0 0 0
b
Mayo validation cohort
PFS Probability (%)
p = 0.12
Time
ICI-mono, CDKN2A mutation
ICI-chemo, CDKN2A mutation
ICI-mono, CDKN2A deletion
ICI-chemo, CDKN2A deletion
Number at risk
2 2 2 2 2 1 0
8 2 0 0 0 0 0
3 1 0 0 0 0 0
7 1 1 1 1 1 0
c
MDACC-validation cohort
PFS Probability (%)
p = 0.02
0
10
20
30
40
Time
ICI-mono, CDKN2A mutation
ICI-chemo, CDKN2A mutation
ICI-mono, CDKN2A deletion
ICI-chemo, CDKN2A deletion
Number at risk
16 3 2 1 0
11 3 1 1 0
4 2 1 0 0
1 0 0 0 0

Supplementary Figure 19. **Association between *CDKN2A* alteration type, treatment strategy (ICI-mono: immune checkpoint inhibitor monotherapy; ICI-chemo: immune checkpoint inhibitor with concurrent chemotherapy), and progression-free survival (PFS)**. **a,** MDACC-primary cohort; **b,** Mayo-validation cohort; **c,** MDACC-validation cohort. P values were calculated using log-rank analysis. Source data are provided as a Source Data file.

a

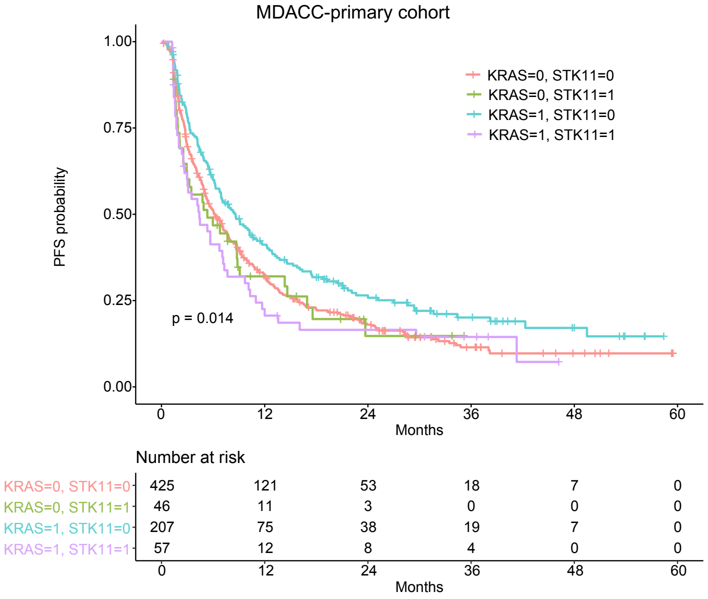
MDACC-primary cohort
PFS probability
1.00
0.75
0.50
0.25
0.00
KRAS=0, STK11=0
KRAS=0, STK11=1
KRAS=1, STK11=0
KRAS=1, STK11=1
p = 0.014
0
12
24
36
48
60
Months
Number at risk
KRAS=0, STK11=0 425 121 53 18 7 0
KRAS=0, STK11=1 46 11 3 0 0 0
KRAS=1, STK11=0 207 75 38 19 7 0
KRAS=1, STK11=1 57 12 8 4 0 0
0 12 24 36 48 60
Months

b

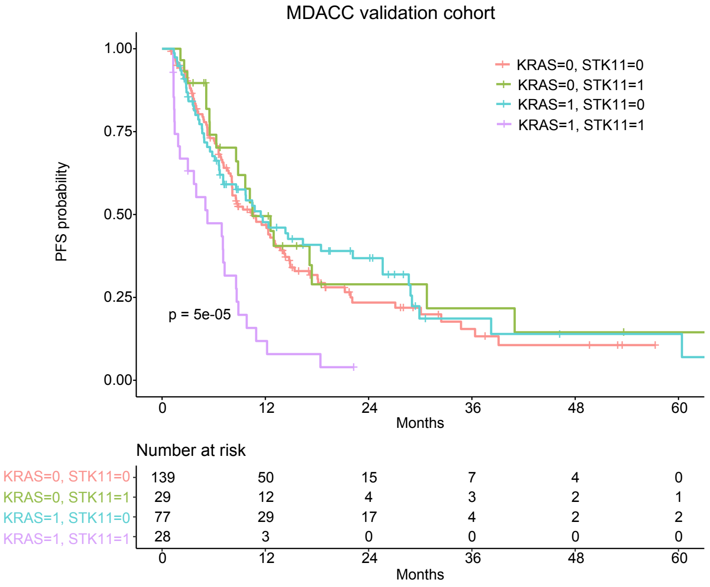
MDACC validation cohort
PFS probability
1.00
0.75
0.50
0.25
0.00
KRAS=0, STK11=0
KRAS=0, STK11=1
KRAS=1, STK11=0
KRAS=1, STK11=1
p = 5e-05
0
12
24
36
48
60
Months
Number at risk
KRAS=0, STK11=0 139 50 15 7 4 0
KRAS=0, STK11=1 29 12 4 3 2 1
KRAS=1, STK11=0 77 29 17 4 2 2
KRAS=1, STK11=1 28 3 0 0 0 0
0 12 24 36 48 60
Months

Supplementary Figure 20. **Impact of *STK11* and *KRAS* co-mutations on progression-free survival (PFS) . a,** MDACC-primary cohort and **b,** MDACC-validation cohort; p values were calculated using log-rank analysis. Source data are provided as a Source Data file.

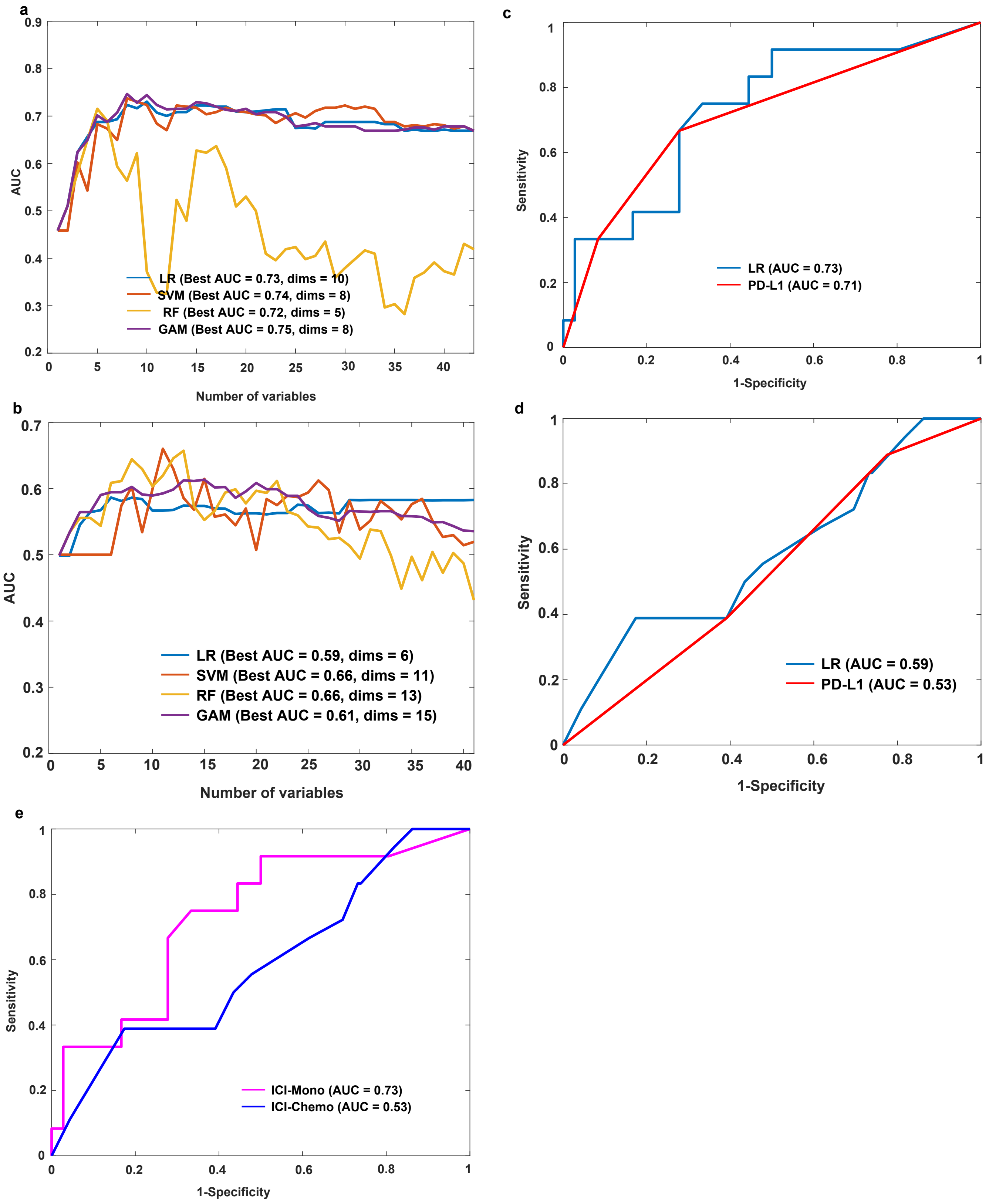
a
AUC
Number of variables
LR (Best AUC = 0.73, dims = 10)
SVM (Best AUC = 0.74, dims = 8)
RF (Best AUC = 0.72, dims = 5)
GAM (Best AUC = 0.75, dims = 8)
b
AUC
Number of variables
LR (Best AUC = 0.59, dims = 6)
SVM (Best AUC = 0.66, dims = 11)
RF (Best AUC = 0.66, dims = 13)
GAM (Best AUC = 0.61, dims = 15)
c
Sensitivity
1-Specificity
LR (AUC = 0.73)
PD-L1 (AUC = 0.71)
d
Sensitivity
1-Specificity
LR (AUC = 0.59)
PD-L1 (AUC = 0.53)
e
Sensitivity
1-Specificity
ICI-Mono (AUC = 0.73)
ICI-Chemo (AUC = 0.53)

Supplementary Figure 21. **Performance of the clinicogenomic predictive models of 3-months progression in the MDACC-validation cohort (n=393) in (a, c) immune checkpoint inhibitor (ICI) monotherapy (ICI-mono) and (b, d) ICI with concurrent chemotherapy (ICI-chemo) treated patients**. **a-b,** area under the curve (AUC) values with increasing features and different models (LR - logistic regression, SVM - support vector machines, RF - random forest, GAM - generalized additive model) in **a,** ICI-mono and **b,** ICI-chemo; **c-d** Receiver operator characteristic (ROC) curves for the best performing LR model vs PD-L1 alone in **c,** ICI-mono and **d,** ICI-chemo; **e,** ROC curves for the best performing LR models in ICI-mono vs ICI-chemo. dims: number of features for best AUC value. Source data are provided as a Source Data file.

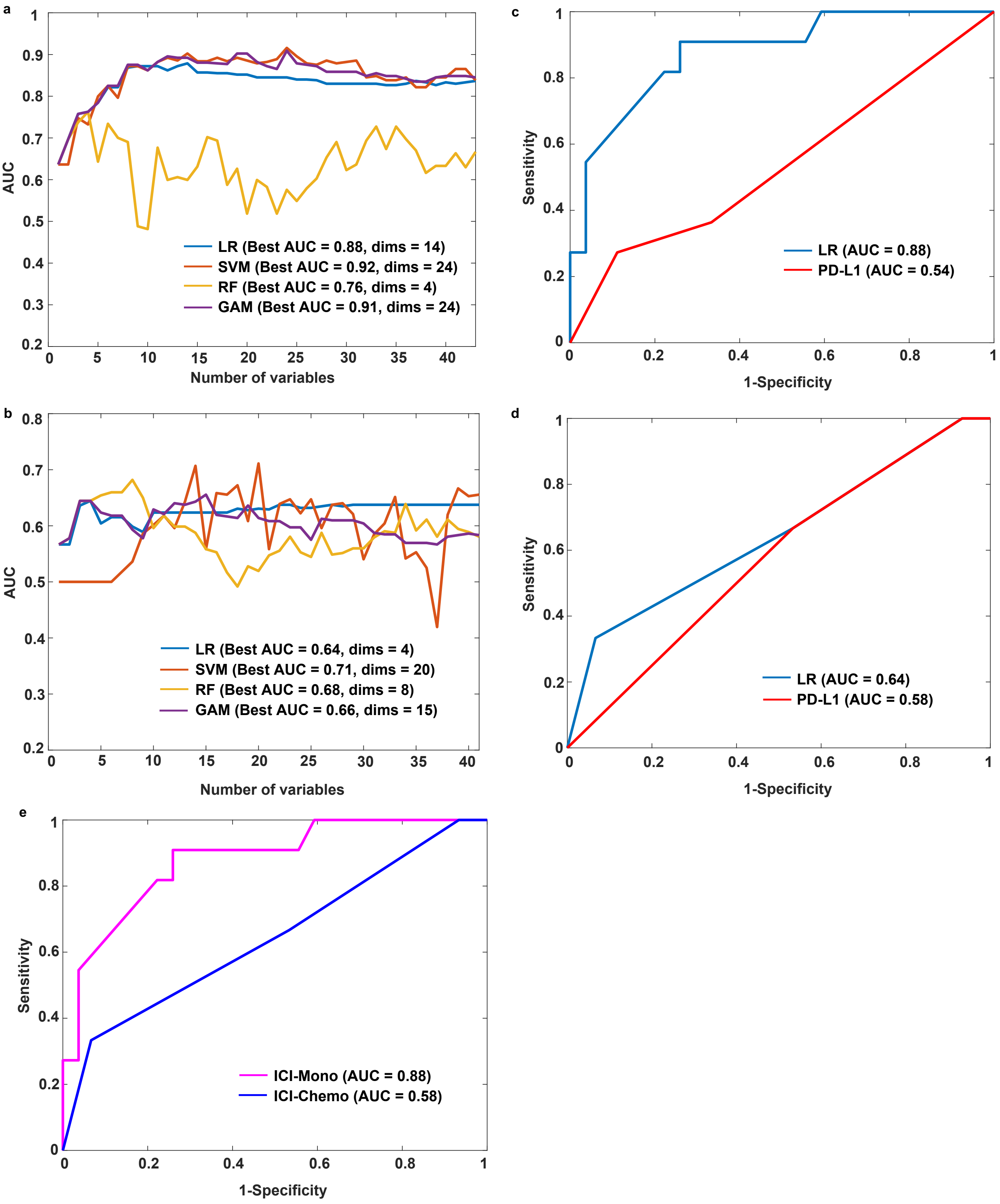
a
AUC
Number of variables
LR (Best AUC = 0.88, dims = 14)
SVM (Best AUC = 0.92, dims = 24)
RF (Best AUC = 0.76, dims = 4)
GAM (Best AUC = 0.91, dims = 24)
b
AUC
Number of variables
LR (Best AUC = 0.64, dims = 4)
SVM (Best AUC = 0.71, dims = 20)
RF (Best AUC = 0.68, dims = 8)
GAM (Best AUC = 0.66, dims = 15)
c
Sensitivity
1-Specificity
LR (AUC = 0.88)
PD-L1 (AUC = 0.54)
d
Sensitivity
1-Specificity
LR (AUC = 0.64)
PD-L1 (AUC = 0.58)
e
Sensitivity
1-Specificity
ICI-Mono (AUC = 0.88)
ICI-Chemo (AUC = 0.58)

Supplementary Figure 22. **Performance of the clinicogenomic predictive models of 3-months progression in the Mayo validation cohort (n=89) in (a, c) immune checkpoint inhibitor (ICI) monotherapy (ICI-mono) and (b, d) ICI with concurrent chemotherapy (ICI-chemo) treated patients**. **a-b,** area under the curve (AUC) values with increasing features and different models (LR - logistic regression, SVM - support vector machines, RF - random forest, GAM - generalized additive model) in **a,** ICI-mono and **b,** ICI-chemo; **c-d** Receiver operator characteristic (ROC) curves for the best performing LR model vs PD-L1 alone in **c,** ICI-mono and **d,** ICI-chemo; **e,** ROC curves for the best performing LR models in ICI-mono vs ICI-chemo. dims: number of features for best AUC value. Source data are provided as a Source Data file.

Supplementary Table 1, Clinical associations between *KRAS* mutant and *KRAS* wild-type patients. P values were calculated using chi-square analysis. ICI, immune checkpoint inhibitor; LUAD, lung adenocarcinoma; LUSC, lung squamous cell carcinoma; ICI-mono, immune checkpoint inhibitor monotherapy; ICI-chemo, immune checkpoint inhibitor with concurrent chemotherapy. Source data are provided as a Source Data file.

Parameters, n (%)	*KRAS* negative (n=480)	*KRAS* positive (n=255)	p value
Age at ICI started			
18-65	244 (68)	117 (32)	0.201
> 65	236 (63)	138 (37)	
Gender			
Male	293 (73)	110 (27)	3.438e-06
Female	187 (56)	145 (44)	
Tobacco use			
Never	118 (83)	24 (17)	8.43e-07
Former	277 (59)	191 (41)	
Current	85 (68)	40 (32)	
Histology			
LUAD	364 (61)	229 (39)	4.53e-08
LUSC	94 (90)	10 (10)	
Others	22 (58)	16 (42)	
PD-L1 expression			
< 1%	134 (71)	54 (29)	0.075*
1-49%	128 (68)	61 (32)	
≥50%	99 (60)	66 (40)	
Unknown	119 (62)	74 (38)	
Treatment			
ICI-mono	314 (67)	152 (33)	0.120
ICI-chemo	166 (62)	103 (38)	
Line of ICI			
First line	279 (63)	167 (37)	0.060
Second line	157 (68)	75 (32)	
> 2nd line	44	13	
Metastatic status at ICI start			
IVA	199 (66)	104 (34)	0.860
IVB	281 (65)	151 (35)	
Liver metastasis at ICI start			
No	417 (66)	215 (34)	0.341
Yes	63 (61)	40 (39)	
Brain metastasis at ICI start			
No	339 (65)	183 (35)	0.746
Yes	141 (66)	72 (34)	

*Cases with PD-L1 unknown were excluded for analysis.
