# Supplementary material for: Efficacy and clinicogenomic correlates of response to immune checkpoint inhibitors alone or with chemotherapy in non-small cell lung cancer
Source: Nat Commun. 2023 Feb 8;14:695. doi: 10.1038/s41467-023-36328-z (PMC9908867; doi:10.1038/s41467-023-36328-z)
Supplement: Supplementary file 3 — Reporting Summary [file 41467_2023_36328_MOESM3_ESM.pdf]

nature portfolio

Corresponding author(s): Natalie Vokes

Last updated by author(s): Dec 9, 2022

Reporting Summary

Nature Portfolio wishes to improve the reproducibility of the work that we publish. This form provides structure for consistency and transparency in reporting. For further information on Nature Portfolio policies, see our Editorial Policies and the Editorial Policy Checklist.

Statistics

For all statistical analyses, confirm that the following items are present in the figure legend, table legend, main text, or Methods section.

n/a	Confirmed	
☐	☒	The exact sample size (n) for each experimental group/condition, given as a discrete number and unit of measurement
☐	☒	A statement on whether measurements were taken from distinct samples or whether the same sample was measured repeatedly
☐	☒	The statistical test(s) used AND whether they are one- or two-sided<br>*Only common tests should be described solely by name; describe more complex techniques in the Methods section.*
☐	☒	A description of all covariates tested
☐	☒	A description of any assumptions or corrections, such as tests of normality and adjustment for multiple comparisons
☐	☒	A full description of the statistical parameters including central tendency (e.g. means) or other basic estimates (e.g. regression coefficient) AND variation (e.g. standard deviation) or associated estimates of uncertainty (e.g. confidence intervals)
☐	☒	For null hypothesis testing, the test statistic (e.g. F, t, r) with confidence intervals, effect sizes, degrees of freedom and P value noted<br>*Give P values as exact values whenever suitable.*
☒	☐	For Bayesian analysis, information on the choice of priors and Markov chain Monte Carlo settings
☐	☒	For hierarchical and complex designs, identification of the appropriate level for tests and full reporting of outcomes
☒	☐	Estimates of effect sizes (e.g. Cohen's d, Pearson's r), indicating how they were calculated

Our web collection on statistics for biologists contains articles on many of the points above.

Software and code

Policy information about availability of computer code

Data collection	The study does not utilize software or code for data collection.
Data analysis	No custom software was used to analyze the data in this study. A list of commercial softwares used in this study are listed below and separated by coma: R software (Version 4.0.3), MATLAB (Version R2021a), Python (Version 2.7.18), along with the R packages survminer (0.4.9), survival (3.2.13), MASS (7.3.54), dplyr (1.0.7), subtee (1.0.1), adjustedCurves (0.9.0). Reference scripts and data to reproduce the 3-month progression model are deposited at https://github.com/nvokes/GEMINI_IO/tree/main

For manuscripts utilizing custom algorithms or software that are central to the research but not yet described in published literature, software must be made available to editors and reviewers. We strongly encourage code deposition in a community repository (e.g. GitHub). See the Nature Portfolio guidelines for submitting code & software for further information.

Data

Policy information about availability of data

All manuscripts must include a data availability statement. This statement should provide the following information, where applicable:

- Accession codes, unique identifiers, or web links for publicly available datasets
- A description of any restrictions on data availability
- For clinical datasets or third party data, please ensure that the statement adheres to our policy

Deidentified clinical data for patients in the MDACC-primary cohort reported in this study are available in Source data for Table 1 and 2. Deidentified molecular data for patients in the MDACC-primary cohort are available in Source data for Supplementary Fig 2. The raw sequencing data are protected and are not available due to

privacy laws. Additionally, anonymized data and the input for the predictive models are available at GitHub (https://github.com/nvokes/GEMINI_IO/tree/main). The remaining data are available within the Article, Source Data, Supplementary Information, and Data files. Source data are provided with this paper.

Human research participants

Policy information about studies involving human research participants and Sex and Gender in Research.

Reporting on sex and gender	This retrospective study is a gender-based analysis. Gender information was collected from medical record and used as a main variable in all subgroup analysis.
Population characteristics	The detailed patient characteristics are summarized into Table 1, Supplementary Data 1, and Supplementary Figure 1.
Recruitment	All patient data used in this study were obtained from the GEMINI database and external institutions with no selection bias.
Ethics oversight	MDACC Institutional Review Board (IRB) approval was obtained. The protocols approved for this study are listed below:<br>Data collection protocol: PA13-0589: GEMINI-Moonshot Project: A prospective database for patients with lung cancer incorporating collection of tissue and clinical information<br>Data use protocol: PA16-0061: Comprehensive analysis of lung cancer patients' clinical information collected into GEMINI database<br>This study was also approved by the IRB at Mayo Clinic and Massachusetts General Hospital (MGH), and all patients provided written informed consent.

Note that full information on the approval of the study protocol must also be provided in the manuscript.

Field-specific reporting

Please select the one below that is the best fit for your research. If you are not sure, read the appropriate sections before making your selection.

☒ Life sciences ☐ Behavioural & social sciences ☐ Ecological, evolutionary & environmental sciences

For a reference copy of the document with all sections, see nature.com/documents/nr-reporting-summary-flat.pdf

Life sciences study design

All studies must disclose on these points even when the disclosure is negative.

Sample size	No sample-size calculation was performed. The number of patients was determined based on the inclusion criteria, data availability and patient enrollment.
Data exclusions	No data were excluded from this study.
Replication	Replicates were not feasible due to the retrospective cohort, but cross-validation was performed to simulate replicates for model testing.
Randomization	This was a retrospective study, and all qualified patients were selected prior to the date: MDACC GEMINI database - January 2022; Mayo validation cohort - January 2020; MGH validation cohort - January 2020. Thus, randomization of patients was not applicable.
Blinding	This was a retrospective study, and all qualified patients were selected prior to the date: MDACC GEMINI database - January 2022; Mayo validation cohort - January 2020; MGH validation cohort - January 2020. Thus, blinding of patients was not applicable.

Reporting for specific materials, systems and methods

We require information from authors about some types of materials, experimental systems and methods used in many studies. Here, indicate whether each material, system or method listed is relevant to your study. If you are not sure if a list item applies to your research, read the appropriate section before selecting a response.

March 2021

Materials & experimental systems

n/a	Involved in the study
☒	☐ Antibodies
☒	☐ Eukaryotic cell lines
☒	☐ Palaeontology and archaeology
☒	☐ Animals and other organisms
☒	☐ Clinical data
☒	☐ Dual use research of concern

Methods

n/a	Involved in the study
☒	☐ ChIP-seq
☒	☐ Flow cytometry
☒	☐ MRI-based neuroimaging
