# Supplementary material for: Efficacy and clinicogenomic correlates of response to immune checkpoint inhibitors alone or with chemotherapy in non-small cell lung cancer
Source: Nat Commun. 2023 Feb 8;14:695. doi: 10.1038/s41467-023-36328-z (PMC9908867; doi:10.1038/s41467-023-36328-z)
Supplement: Supplementary file 4 — Description of Additional Supplementary Files [file 41467_2023_36328_MOESM4_ESM.docx]

**Description of Additional Supplementary Files**

Supplementary Data 1

Description: Clinicopathologic features of patients in the MDACC (n=393) and Mayo (n=89) validation cohorts. P values were calculated using chi-square analysis.

Supplementary Data 2

Description: Genomic data in the MDACC-Primary cohort.

Supplementary Data 3

Description: Genomic data in the MDACC-Validation cohort.

Supplementary Data 4

Description: Genomic data in the Mayo cohort.

Supplementary Data 5

Description: Univariate analysis of clinical features on MDACC primary cohort (n=1,133)

Supplementary Data 6

Description: The ranking score for ICI-mono cohort with PD-L1 and molecular data available (n=312)

Supplementary Data 7

Description: The ranking score for ICI-chemo cohort with PD-L1 and molecular data available (n=230)

Supplementary Data 8

Description: Gene panel used in tissue and blood
